# Machine learning for identification of silylated derivatives from mass spectra

**DOI:** 10.1186/s13321-022-00636-1

**Published:** 2022-09-15

**Authors:** Milka Ljoncheva, Tomaž Stepišnik, Tina Kosjek, Sašo Džeroski

**Affiliations:** 1 Department of Environmental Sciences, Jozef Stefan Institute, Jamova 39, 1000 Ljubljana, Slovenia; 2 Department of Knowledge Technologies, Jozef Stefan Institute, Jamova 39, 1000 Ljubljana, Slovenia; 3 Jozef Stefan International Postgraduate School, Jamova 39, 1000 Ljubljana, Slovenia

**Keywords:** Silylation, Derivative, Identification, Machine learning, Mass spectrometry, Molecular fingerprint, Prediction

## Abstract

**Motivation:**

Compound structure identification is using increasingly more sophisticated computational tools, among which machine learning tools are a recent addition that quickly gains in importance. These tools, of which the method titled Compound Structure Identification:Input Output Kernel Regression (CSI:IOKR) is an excellent example, have been used to elucidate compound structure from mass spectral (MS) data with significant accuracy, confidence and speed. They have, however, largely focused on data coming from liquid chromatography coupled to tandem mass spectrometry (LC–MS).

Gas chromatography coupled to mass spectrometry (GC–MS) is an alternative which offers several advantages as compared to LC–MS, including higher data reproducibility. Of special importance is the substantial compound coverage offered by GC–MS, further expanded by derivatization procedures, such as silylation, which can improve the volatility, thermal stability and chromatographic peak shape of semi-volatile analytes. Despite these advantages and the increasing size of compound databases and MS libraries, GC–MS data have not yet been used by machine learning approaches to compound structure identification.

**Results:**

This study presents a successful application of the CSI:IOKR machine learning method for the identification of environmental contaminants from GC–MS spectra. We use CSI:IOKR as an alternative to exhaustive search of MS libraries, independent of instrumental platform and data processing software. We use a comprehensive dataset of GC–MS spectra of trimethylsilyl derivatives and their molecular structures, derived from a large commercially available MS library, to train a model that maps between spectra and molecular structures. We test the learned model on a different dataset of GC–MS spectra of trimethylsilyl derivatives of environmental contaminants, generated in-house and made publicly available. The results show that 37% (resp. 50%) of the tested compounds are correctly ranked among the top 10 (resp. 20) candidate compounds suggested by the model. Even though spectral comparisons with reference standards or de novo structural elucidations are neccessary to validate the predictions, machine learning provides efficient candidate prioritization and reduction of the time spent for compound annotation.

**Supplementary Information:**

The online version contains supplementary material available at 10.1186/s13321-022-00636-1.

## Introduction

Growing awareness of the environmental impact on human health has increased interest into the environmental chemical space of the human exposome, that consists of the multitude of structurally and toxicologically diverse synthetic and naturally occurring compounds [1–3]. This has turned the annotation of contaminants of emerging concern (CEC) into a task of utmost importance [4–6], as it can provide valuable knowledge about their identity, accumulation, degradation and transformation patterns, exposure pathways and toxicity. Among the multitude of chemical, biological and toxicity estimation methods, chromatography coupled to MS methods has become the essential analytical tool for thorough CEC annotation. Employment of strongly consolidated, targeted, suspect screening and non-targeted screening strategies requires the use of data processing software, cheminformatics tools, ever-growing compound databases (DBs), MS libraries (MSLs) and computational MS workflows for assignment of chemical identities to MS signals.

In its beginning, MS-based high throughput exposome exploration involved manual determination of compound’s molecular weight (M_W_), computation of a molecular formula (MF) and then search against data repositories for candidates. Different data resources have been used for this purpose, including user-generated specified suspect lists (e.g. [7, 8]), specialized lists compiled by, e.g., the US EPA’s Distributed Structure-Searchable Toxicity (DSSTox) database [9] and environmental communities such as the NORMAN Network [10]. Medium-sized DBs contain tens to hundreds of thousands of compounds (e.g., US EPA’s Comptox Chemistry Dashboard (CCD) [11], ContaminantDB [12], the Toxin and Toxin Target Database (T3DB) [13], the Exposome Explorer [14]), while the most comprehensive chemical repositiories, such as PubChem [15] and Chemspider [16] can contain over 100 million compounds. The latter are the most frequently exploited sources. They offer an exceptionally wide chemical space, hence a simple exact mass or MF search rapidly turns into a non-target identification challenge, often with hundreds to thousands of hits [7, 17]. Later, MSLs were introduced to obtain rapid tentative identifications at relatively high confidence [18]. Many MSLs either contain predominantly LC–MS data (e.g., the Human Metabolome Database 4.0 [19], METLIN [20], MassBank [21], mzCloud [22]), GC–MS data (e.g., the Golm Metabolome Database (GMD) [23], the Fiehn Library [24]), or both (e.g., National Institute of Standards and Technology (NIST) Mass Spectral Library [25] and Wiley Registry [26]). Compounds are identified by comparing experimentally acquired and reference MSL spectra using versatile spectral similarity functions. Yet even nowadays, in the era of their substantial increase in size and comprehensiveness, MSLs cover only a fraction of the exposomics-relevant chemical information, as inclusion of newly identified CEC is inherently limited by the availability of reference standards, the relative youth and the lack of their standardization [27]. This coverage is even poorer for silyl derivatives, with very few MSLs [23–25] containing their MS spectra.

In the last decade, compound structure identification (CSI) based on compound DBs and MSLs has been replaced by numerous cheminformatics methods [28]. These methods perform CSI by either determining the exact mass or MF, by using a predefined exact mass or MF, or by converting the structural information inherent to MS data, including the presence of specific substructures, functional groups or complete fragmentation pathways, into a computationally more convenient “third format”. Here, “third format” representation of the structural information contained in MS spectra includes more computationally manageable formats, such as fragmentation trees, mass spectral trees (for multi-stage MS data, MS^n^), and molecular fingerprints (MFP), all which include structural information that can be extracted from an MS spectrum and further processed. Based on this third format, the cheminformatics approaches perform exhaustive interrogation/search of MSLs or compound DBs to create candidate sets, from which, according to (sub)structural similarity (possibly accompanied with other criteria, such as chromatographic behaviour, energy, data source, environmental behavior and toxicity related criteria and/or complementary information), most probable candidates are prioritized and ranked [28, 29]. Among these approaches, those based on machine learning (ML) have offered highest accuracy, confidence and speed in performing the CSI task [7, 29, 30].

Revolutionary breakthroughs in the technological development of GC/LC coupled to MS (GC–MS and LC–MS, respectively), especially high resolution/accurate mass—mass spectrometry (HR/AM-MS), allow for measuring hundreds to thousands of chemical features, represented by MS signals, in a single complex sample [6, 31]. LC–MS analytical platforms are considered “the golden standard’’ in exposomics research, shadowing the GC–MS analytical platforms. Despite offering highly efficient, sensitive and reproducible analysis with relatively modest cost and substantial compound coverage, GC–MS is a somewhat underestimated source of valuable complementary analytical data in CEC annotation [32]. The ultimately predominant ionisation method for the acquisition of GC–MS spectra is electron impact (EI) ionisation, along with the less frequently used chemical ionization. The great reproducibility of EI spectra, following predictable and thoroughly studied fragmentation patterns and broad internal energy distribution, promises highly accurate, yet not thoroughly explored, instrument-independent data for CSI. Even less explored is the identification of semi-volatile and thermolabile compounds using the MS data of their silylated derivatives, mainly trimethylsilyl (TMS) or *tert*-butyl dimethylsilyl (TBDMS) derivatives. While being useful in greatly enhancing the compounds’ chromatographic and mass spectrometric characteristics, the derivatization may complicate peak annotations due to sometimes incomplete derivatization processes, with formation of multiple and/or partially derivatized compounds. Moreover, TMS and TBDMS derivatives and their MS spectra are poorly represented in compound DBs and MSLs, and, accordingly, they are not readily identified using the traditional CSI approaches of compound DB(s) and/or MSL(s) search. While cheminformatics CSI approaches are expected to solve this task as well, they have been almost exclusively developed and tested using electrospray LC-(ESI)-MS/MS data and are yet to be challenged against GC-EI-MS data.

This paper presents the first application of a machine learning approach, named Compound Structure Identification:Input Output Kernel Regression (CSI:IOKR), for the identification of CEC silyl derivatives using GC-EI-MS spectra. First, we generate two unique collections of GC-EI-MS spectra of TMS derivatives: a collection curated from the NIST 17 Mass Spectral Library that is used to train a model with CSI:IOKR and a collection of GC-EI-MS spectra experimentally acquired in our laboratory that is used to test the model. Second, we evaluate the performance of the CSI:IOKR model in identifying CEC silyl derivatives. Note that we have generated our own test data (thus using different sources for the training and testing data) for two reasons: (1) to maximize the size of the training data, and (2) to obtain better estimates of the performance of the model in its intended use scenario, i.e., for identification of CEC compounds through their silyl derivatives, on unseen data. We also investigate how identification performance depends on several factors, including the filtering of the training dataset, the overlap between compounds in the training and the test datasets, and the post-acquisition processing of the test dataset. The CSI:IOKR approach reaches satisfactory identification performance for TMS derivatives, both within and outside the training dataset, indicating its potential for use in GC–MS based annotation of contaminants.

## Related work

**Fig. 1:**
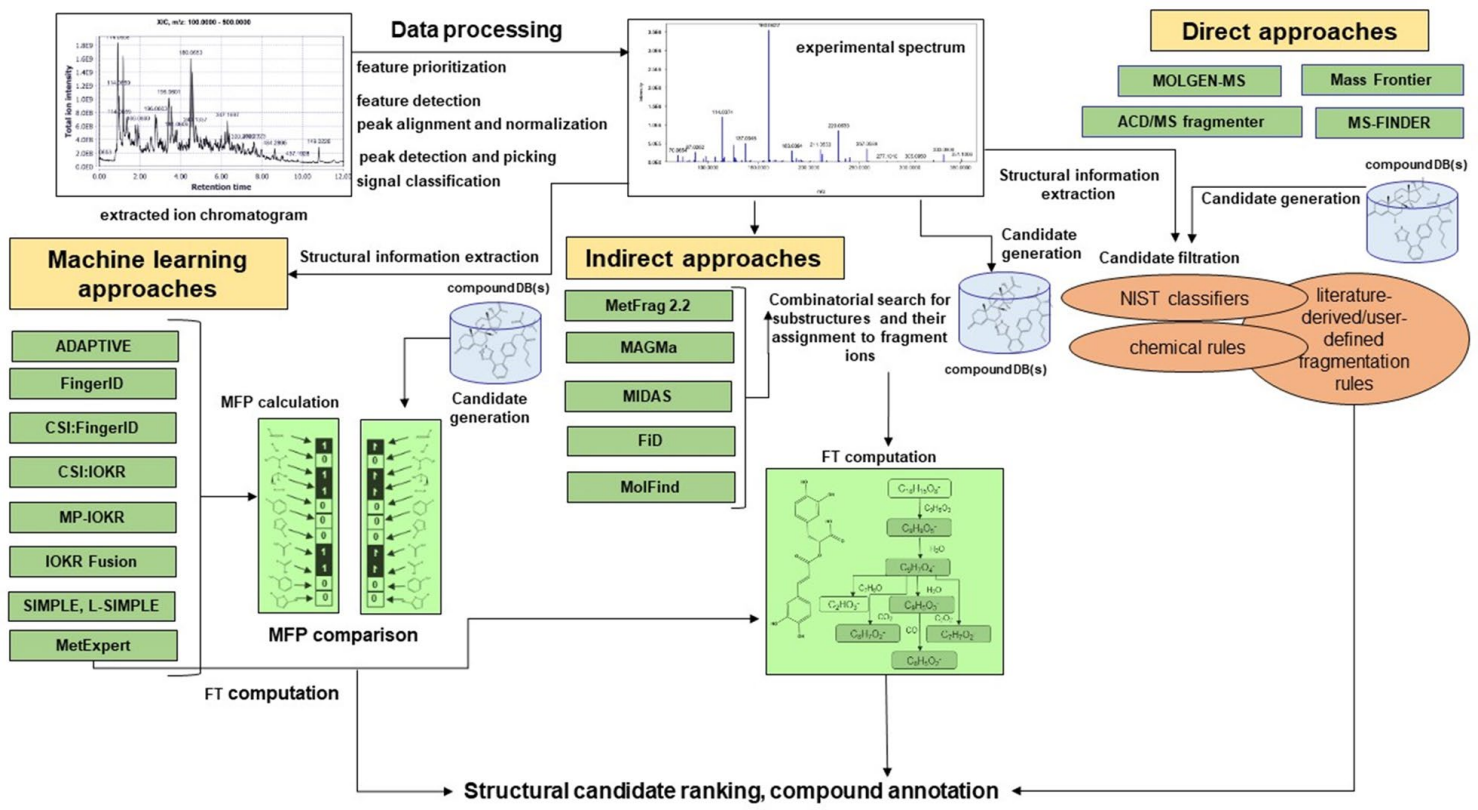
An overview of direct, indirect and joint approaches for compound structure identification. Adapted from Ljoncheva et al. [28]

The field of cheminformatics-assisted compound structure identification (CSI) has grown intensively over the last two decades, developing three groups of approaches (Fig. 1). The simplest ones are direct approaches, such as Mass Frontier [33], ACD/MS Fragmenter [34], MOLGEN-MS [35] and MS-FINDER [36], that extract and use structural information directly from the MS spectra, represented as a set of *m/z* values of molecular ions, relative abundances of isotopologues, given the MF or fragment ions. Indirect approaches include the combinatorial fragmentation methods, e.g., FiD [37], MetFrag 2.2 [38], MAGMa [39], MolFind [40] and MIDAS [41]. Approaches from the third group, including MetExpert [42], FingerID [43], CSI:FingerID [44], CSI:IOKR [45], magnitude-preserving IOKR (MP-IOKR) [46], IOKRFusion [47], SIMPLE and L-SIMPLE [48] and ADAPTIVE [49], rely on the use of machine learning. The third group utilizes the alternative concept of in silico spectral prediction, i.e., prediction of two dimensional (*m/z* and intensity) EI-MS (CFM-ID [50], NEIMS [51]) or ESI–MS/MS spectra (CFM-ID [52], ISIS [53]) by simulating fragmentation for a defined compound candidate set and performing CSI by comparing the measured and the in silico predicted MS/MS spectra [28]. The cutting-edge CSI approaches are more thoroughly described in recent reviews [28, 30].

In their core, the indirect “third-format” ML approaches transform the MS structural information into “third formats”, such as MFP, molecular descriptors, or their combination, that have higher discriminatory power to reflect structural similarity and therefore lead to more accurate and confident compound structure identification. The ice-breaking ML-based approach is FingerID [43], that in a first step uses the probability product kernel (PPK) [54] directly computed from MS spectra and runs support vector machines to perform MFP predictions. In the second step, it ranks candidates from DB-derived sets according to their similarity to the predicted MFP. This method is mainly based on the information from the individual spectral peaks and ignores their interactions. The follow-up approach, CSI:FingerID [44], uses MS spectra and fragmentation trees to calculate multiple kernels combined via multiple kernel learning [55], resulting in improved predictive performance. Its disadvantage is in the long running times due to the “one-at-a-time” spectrum processing approach and computationally heavy conversions of MS spectra into fragmentation trees. The CSI:IOKR approach [45] learns mappings from MS spectra to MFP using multiple input kernels to encode similarities in the input space (MS spectra) and output kernels for encoding similarities in the output space (MFP). It predicts all components of a MFP simultaneously, resulting in a faster one-step approach. Further efforts to preserve the discrepancy between compounds in the input space, and between candidates in the output space, as well as incorporate candidate ranking information in the learning phase resulted in the development of MP-IOKR [46], with improved compound identification accuracy as compared to CSI:IOKR. The latest method in the IOKR series, IOKRFusion [47] is a score aggregation method that cobmines 60 IOKR models and 60 IOKR reverse models that learn the mapping of molecular structures into the MS/MS feature space rather than the output feature space. Finally, MFP are combined with ML prediction of retention indices and compound substructures, in silico derivatization of DBs, and metabolite-likeness evaluation in the MetExpert approach [56].

The ultimate ML-based “third-format” approaches exchange either the fixed, redundant MFP with novel, non-redundant, data-driven and specific molecular vectors (ADAPTIVE [49]) or multiple kernels with a simpler prediction function, incorporating peak interactions (SIMPLE [48]). The first method combines the learning of a mapping from structures to molecular vectors utilizing message passing neural network with IOKR-based learning of the mapping from MS spectra to molecular vectors. The second method offers performance comparable to that of kernel-based methods at higher prediction speed that is proportional to the number of peaks in the queried spectrum, unlike all aforementioned kernel-based methods [30].

The most recent Critical Assessment of Small Molecule Identification (CASMI) contests (2016 [57] and 2017 [58]) identified the ML-based approaches CSI:FingerID [44], CSI:IOKR [45] and CFM-ID [59] as the most accurate compound structure identification tools, ranking as top1 and among the top10 17 and 34.4% (for CSI:IOKR) and more than 49% of the challenges, respectively. The challenges used LC–ESI–MS/MS spectra of reference standards. Despite their expansive development and excellent performance, the ML-based compound structure identification tools have been seldomly used in CEC research [28]: they have been used in few LC-(ESI)-MS/MS-based studies [8, 60–65], but no GC-EI-MS-based studies. In fact, only three approaches, including MetExpert [42], CFM-ID [50] and NEIMS [51] have been specifically developed to handle GC-EI-MS data, among which only the first one performs the CSI task on GC-EI-MS spectra of TMS and methoxy/TMS derivatives.

## Materials and methods

### Generation of the training dataset

The NIST 17 Mass Spectral Library [66] was selected as reference MSL for the generation of our training dataset. NIST 17 is the most comprehensive selection of GC-EI-MS spectra, containing 306,622 GC-EI-MS spectra of 267,376 compounds. Two of the NIST 17 libraries were searched; the main spectral library (*mainlib*), with 267,376 GC-EI-MS spectra and the replicate library (*replib*), with 39,246 GC-EI-MS spectra that are independent replicates of spectra of compounds contained in *mainlib*. *Replib* is a collection of noisier spectra as compared to *mainlib,* which reflect normally occuring experimental and instrumental response variations and make the training dataset more informative.

The spectral search was performed by using the NIST MS Search Program v.2.3 (NIST, 2017), with two constraints: *name fragment: trimethysilyl* and *elements allowed: Si.* The GC-EI-MS spectra were extracted in .msp file format and subsequently converted into .txt format using the LIB2NIST conversion tool (NIST, 2011), saving the following data for each extracted GC-EI-MS spectrum: name, InChIKey, MF, M_w_, exact mass, CAS number, NIST ID and MS peak list.

**Fig. 2:**
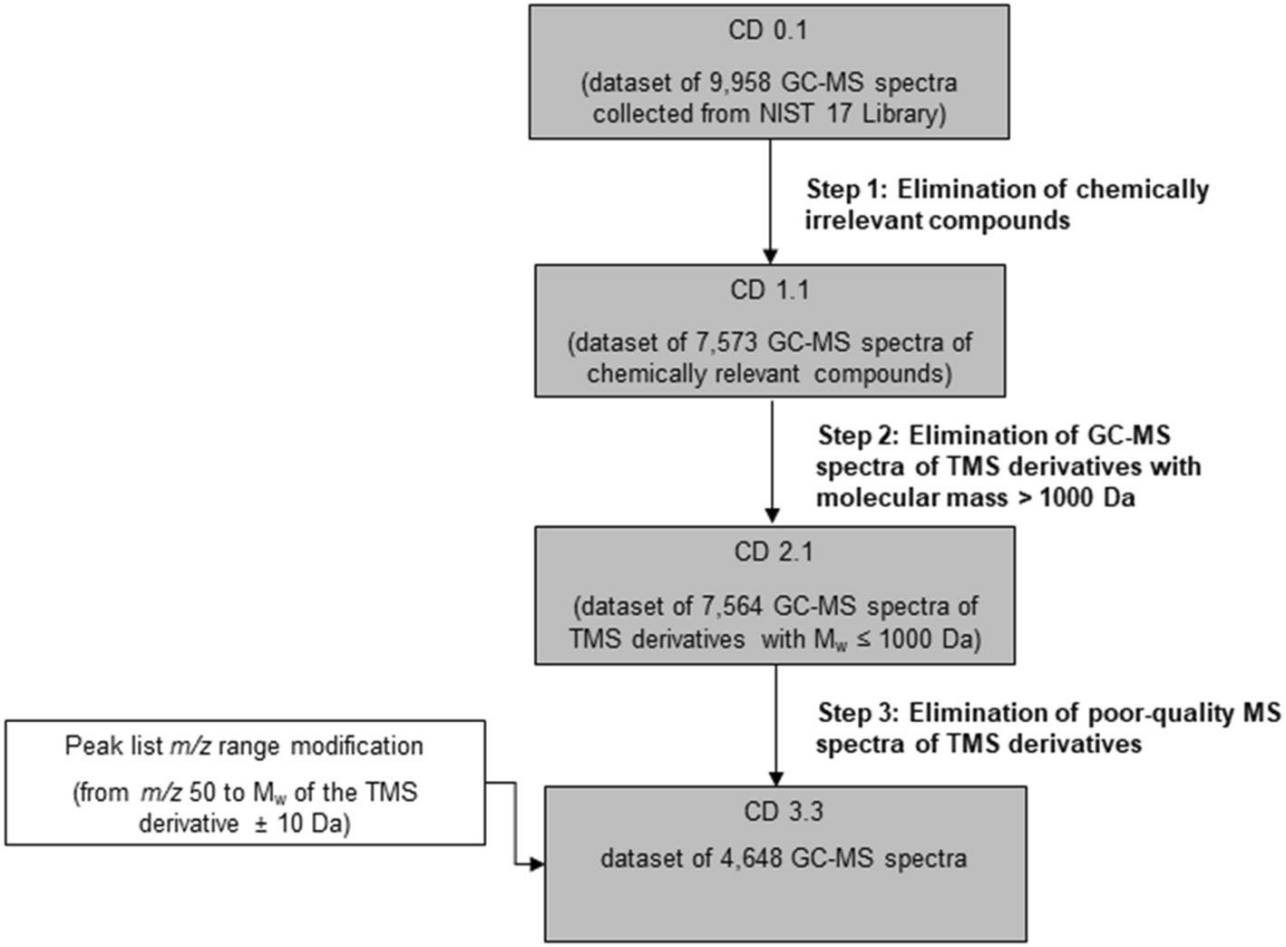
The three-step spectral filtering process. CD 0.1 is the initial GC-EI-MS training dataset curated from NIST, CD 1.1 is the step 1-refined GC-EI-MS dataset, CD 2.1 is the step 1 and 2-refined GC-EI-MS dataset, while CD 3.3 is the final GC-EI-MS dataset that has gone through the three steps of spectral filtering.

The originally extracted NIST 17 entries were filtered by using the three-step approach shown in Fig. 2. The first step involved manual inspection of the spectra to retain only the Si-containing compounds generated as a result of the silylation reaction. The GC-EI-MS spectra of chemically irrelevant Si-containing compounds were removed from the dataset. Here, we set the following structural categories for exclusion:

1. Structures with Si–Si bonds (siloxanes);
2. Structures with C-Si bonds;
3. Structures with O-Si bonds other than hydroxyl/carboxyl-TMS derivatives;
4. Structures with N-Si bonds other than primary/secondary amine-TMS derivatives;
5. Structures with N–O-Si and N–N-Si bonds;
6. Structures with S–Si bonds other than thiol-TMS derivatives;
7. Structures with P-/(O/N/S)-Si bonds;
8. TMS derivatives generated as a result of a rearrangement derivatization reaction;
9. TBDMS derivatives;
10. Mixed TMS and TBDMS derivatives;
11. TMS derivatization agents;
12. TMS derivatives of inorganic compounds and
13. TMS derivatives that contain heavy metals.

As a part of the first data filtering step, we also removed erroneous NIST 17 entries, i.e., those GC-EI-MS spectral entries whose names and structures did not correspond. In the second step, GC-EI-MS spectra of TMS derivatives with *m/z* ≥ 1,000 Da were removed, since such high molecular masses are above the working linear range of most of the mass analyzers used in GC–MS platforms. As a final data filtration step, we used four basic criteria to ensure baseline spectral quality. GC-EI-MS spectra were excluded unless they complied with all of the requirements below:

- GC-EI-MS spectra have to be acquired at the upper *m/z* of at least M_w_ of the derivative + 10 amu;
- GC-EI-MS spectra have to contain the molecular ion [M]^+^ peak and at least one of the isotope peaks, such as the ^13^C isotope peak;
- GC-EI-MS spectra have to contain peaks of fragment ion specific for TMS groups (*m/z* 73, 147, 221 and 295, corresponding to one, two, three and four TMS groups, respectively) and
- GC-EI-MS spectra have to contain at least five fragment ion peaks.

### Generation of the test dataset

#### Chemicals and reagents

From the in-house pool of reference standards, we selected 129 compounds with potential environmental relevance and at least one functional group amenable to TMS derivatization. Preliminary derivatization experiments showed that 100 compounds out of 129 could get successfully derivatized. The list and the basic description of the selected reference standards and other chemicals and reagents used in this study is given in Additional file 1. The compounds are of anthropogenic origin and are potentially bioactive CECs. In order to verify their environmental relevance, the compounds were searched against CCD [11], followed by predicting their environmental properties. US EPA’s Toxicity Estimation Software Tool (T.E.S.T.) [67] was used to predict the common toxicity endopoints: 96 h fathead minnow LC_50_, developmental toxicity and estrogen receptor binding affinity. The Estimation Programs Interface (EPI) Suite^™^ v.4.11 [68] was used to predict the log carbon–water partitioning coefficient (log K_oc_), log octanol–water partitioning coefficient (log K_ow_), water solubility, bioaccumulation factor, bioconcentration factor, biotransformation half-life, half-life in river and half-life in lake, for each of the compounds. To be considered for the test dataset, a compound had to fulfill at least three of the following five criteria, established in accordance with the Regulation (EC) No 1907/2006 of the European Parliament and the Council of 18 December 2006 concerning the Registration, Evaluation, Authorisation and Restriction of Chemicals (REACH), Annex XIII [69]:

1. Positioning (R): the compound is present in the US EPA CCD [11], the most comprehensive repository of eco-exposome constituents;
2. Persistence (P): compound’s half-life in fresh or estuarine water is  > 40 days;
3. Bioaccumulation (B): bioaccumulation factor and/or bioconcentration factor  > 2000, or in absence of such data, logK_ow_ ≥ 5.0;
4. Mobility (M): compound’s water solubility is  ≥ 0.15 mg/L and log K_oc_ is ≤ 4.0, i.e. between −10.0 and 4.0 and
5. EcoToxicity (T): long-term no-observed-effect concentration (NOEC) for marine or freshwater organisms is  < 0.01 mg/L. Here, instead of NOEC, chronic acquatic toxicity (mg/L) for fish, daphnid, and green algae is considered, calculated as the geometric mean of NOEC and lowest observed effect concentration (LOEC).

The results of the Comptox-T.E.S.T and EPI Suite™ predictions are given in Additional file 2.

#### Silylation

The individual stock standard solutions (SSSs) of each compound at the concentration of approximately 150 μg/mL were prepared in EtAc, MeOH or ACN, depending on the solubility of the reference compound (Table 1). The SSSs were kept at + 4 °C and were diluted to prepare working solutions (WSs) at the concentration of 1 μg/mL, which were used within 7 days. TMS derivatives were prepared individually, by mixing 150 µL of a WS with 30 µL of a derivatization agent (MSTFA, BSTFA or BSTFA + 1% TMCS, depending on the derivatization yield determined during the preliminary derivatization experiments). For compounds dissolved in MeOH, the solvent was removed under gentle steam of N_2_ prior to the addition of the derivatization agent, which was followed by reconstitution in 150 µL EtAc and vortexing for 1 min. Derivatization conditions (temperature, time) were selected based on prior optimization, so that compounds were derivatized under either of the following conditions: (1) at 60 °C for 45 min; (2) at 70 °C for 90 min or (3) at 70 °C for 45 min.

**Table 1:** Optimized derivatization and acquisition conditions for CEC-TMS derivatives from the test dataset.

CEC (abbreviations are provided in Additional file 4)			Dissolved in	Derivatization agent and conditions	GC oven programme (see "GC-EI-MS spectra acquisition and dataset compilation" section)
SA LAA SHA	CBD QA	AMP MAMP	MeOH	MSTFA, 60 °C, 45 min	(1)
CBC	THC	CBN		BSTFA + 1% TCMS, 70 °C, 90 min	(2)
11N9THC T3HC 11OHTHC 6-MAM	BZECG LLEU COD	LSER MORPH ERY		BSTFA + 1% TCMS, 70 °C, 45 min	(3)
BA PrPb MePb IBuPb	EtPb BuPb IPrPb	TCS IB BzPb	EtAC	MSTFA, 60 °C, 45 min	(1)
RES CBZ CLA DF 9-HF E1	HPP E2 4-NP E3 NAP EE2	DH-BP BP-8 4,4’-BP SFA KET		BSTFA + 1% TCMS, 70 °C, 90 min	(2)
22BPF BPBP 3M5NC BPAF BPPH 4NC BPF BPFL SYE BPE DHDPE 4-NS BPA PAA PCA BPC 2AA	CA MCA BPB CLP OCA BP26DM AA 17HP BPCL 8-HQ 5AD BPZ 4-OP BD BPS CBDA 6HP	11HT BPAP 4-NG 11HAD H-BP 5-NG ST BHT 6-NG 2APA BPM CAT ET BPP 3MC NX		BSTFA + 1% TCMS, 70 °C, 45 min	(3)
UA			ACN	MSTFA, 60 °C, 45 min	(1)
THCA				BSTFA + 1% TMCS, 70 °C, 90 min	(2)
LTYR				BSTFA + 1% TMCS, 70 °C, 45 min	(3)

#### GC-EI-MS spectra acquisition and dataset compilation

GC-EI-MS spectra were acquired on Agilent 7890B/5977A series GC-MSD (Agilent, USA). Separation was achieved on Agilent DB-5MS UI fused-silica capillary column (30 m × 0.25 mm × 0.25 μm; Agilent, USA). He of 99.99999% purity at the flow rate of 1.2 mL/min was used as a carrier gas. The manifold, ion source and transfer line temperatures were set at 230 °C, 150 °C and 250 °C, respectively. Injections (1 µL) were performed in the splitless mode. Depending upon compound properties, one of the following column oven temperature programs was used: (1) initial temperature 70 ºC (held 1 min), ramped at 15 ºC/min to 280 ºC (held 1 min); total runtime: 16 min; (2) initial temperature 70 ºC (held 1 min), ramped at 20 ºC/min to 240 ºC (held 1 min), at 12 ºC/min to 310 ºC (held 2 min); total runtime: 18.3 min and (3) initial temperature 70 ºC (held 1 min), ramped at 20 ºC/min to 240 ºC (held 1 min), at 12 ºC/min to 310 ºC (held 4 min); total runtime: 20.3 min. The MSD was operated in EI ionization mode (70 eV) by scanning over the mass range of *m/z* 50–800 amu. Mass Hunter Qualitative Analysis v B.07.00 (Agilent, USA) was used to reduce raw instrument data to two-dimensional peak lists (*m/z*, abundance) and to perform background subtraction (BS).

In-between the acquisitions of the derivatized standards, EtAc was run as the solvent check to assess potential background interferences, carryover and sample contamination and was used for background subtraction as a part of the post-acquisition processing of the GC-EI-MS spectra. The test GC-EI-MS dataset was compiled as .txt file that included MF, InChIKey strings, M_w_ and two-dimensional peak lists. Molecular stereochemistry was not considered, since stereoisomers are not readily distinguished by MS.

#### GC-EI-MS spectral similarity analysis and selection

For each TMS-derivative, multiple (≥ 15) GC-EI-MS spectra were generated for the experimental dataset. In order to estimate spectral reproducibility (and therefore the “interchangeability”) of the GC-EI-MS spectra of a TMS derivative, the cosine similarity was calculated. An R script was written to read GC-EI-MS spectra, perform binning in 1.0 Da bins and an intensity N-dimensional vector is constructed in which element v_i_ corresponds to the average peak intensity of all peaks within the bin. The cosine similarity c between spectra v and u was calculated as the dot product of the two vectors divided by the product of their norms (Eq. 1):

1 $$c = \frac{{\sum\nolimits_{i} {v_{i} u_{i} } }}{{\left\| v \right\|\left\| u \right\|}}$$

giving values between 0 and 1, with 0 indicating that the spectra share no common peaks and 1 indicating that the spectra are identical. Cosine similarity is calculated in all-against-all manner in both the RAW and BS experimental datasets. The influence of background subtraction on spectral reproducibility and similarity was explored by calculating the cosine similarity between each raw GC-EI-MS spectrum in the RAW dataset and its corresponding background-subtracted GC-EI-MS spectrum in the BS dataset for each TMS derivative. The results are visualized as a separate cosine similarity measure matrix for each TMS derivative. Finally, in order to explore mass spectral similarities of the TMS derivatives included, a consensus spectrum was built from all binned GC-EI-MS spectra for each TMS derivative. Clustering was then performed of the consensus spectra for both RAW and BS datasets using the distance matrices of all against all consensus RAW, and respective BS spectra.

### CSI:IOKR protocol

**Fig. 3:**
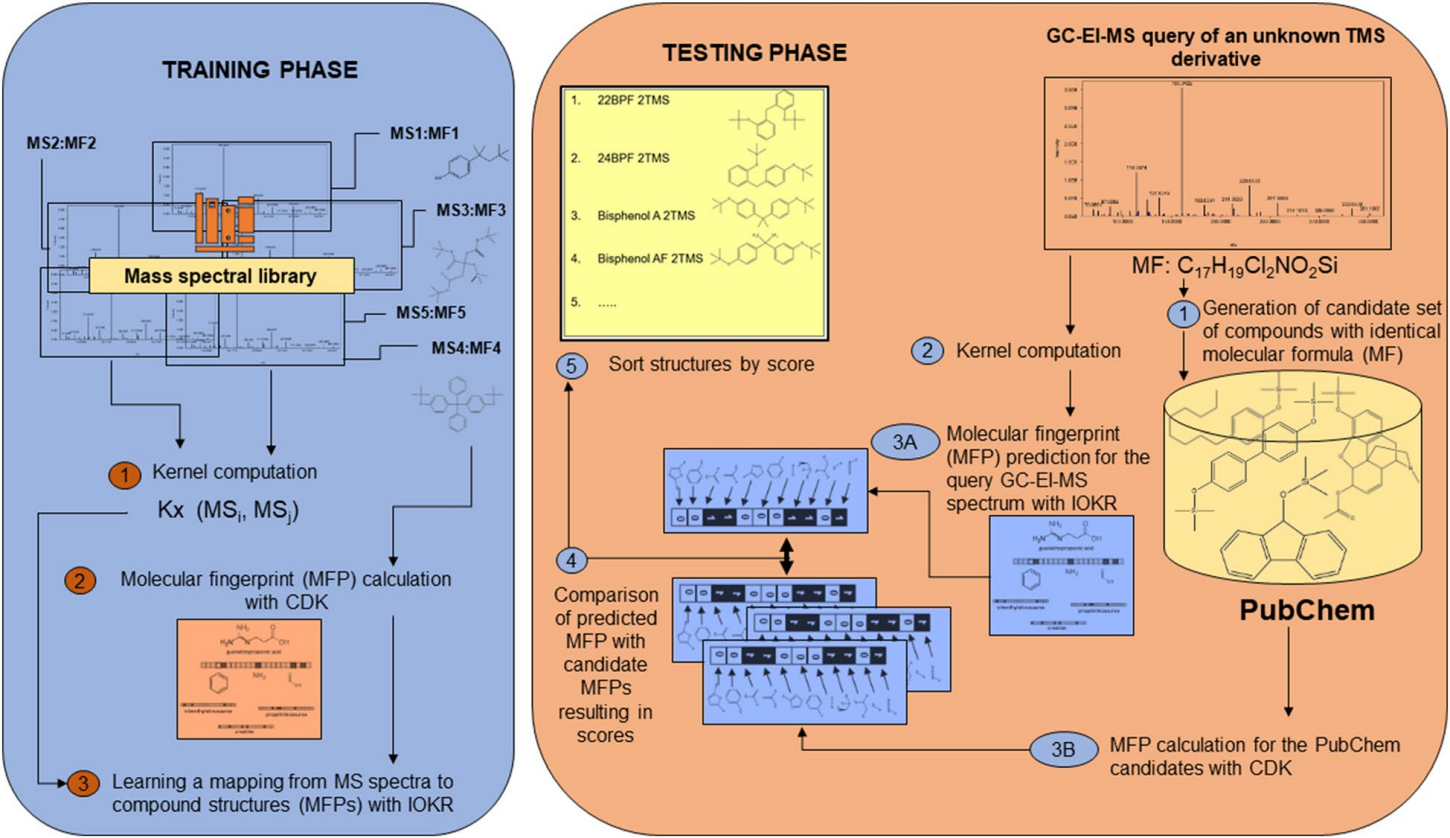
Workflow of the CSI:IOKR protocol that we employ, based on Dührkop et al. [72] and Brouard et al. [45]. The model is trained on a set of GC-EI-MS data from the NIST 17 MSL and the MFPs of the corresponding molecular structures. The PPK kernel is computed for each pair of spectra and MFPs are calculated for each compound from the training NIST 17 MSL dataset in preparation for learning the kernel-based model. The model is tested using GC-EI-MS data of (unknown) TMS derivatives, by calculating the MFPs of candidate compounds with identical MFs, comparing these MFPs with the MFP predicted by the IOKR kernel-based model from the query GC-EI mass spectrum and producing and ordering the list of candidate structures by the similarity of their MFPs with the predicted one.

Identification of TMS derivatives was performed by using a simplified version of CSI:IOKR [45]. The workflow is given in Fig. 3. CSI:IOKR is a kernel-based method, where a kernel function is a positive semi-definite function that measures similarity between two elements [45]. In our study, input kernels measure similarity between MS spectra, while output kernels measure similarity between molecular properties represented as MFPs. The product kernel (PPK) [54] was used as an input kernel, and the linear kernel calculated on MFPs was used as an output kernel. The PPK kernel is computed from MS spectra, by modelling each peak in a spectrum as a normal distribution with two dimensions: *m/z* and intensity, and modelling an MS spectrum as a mixture of normal distributions. The PPK kernel is evaluated by integrating the product between the two corresponding mixture distributions [45]. The kernels were centralized and normalized. The strength of regularization for IOKR was determined with internal cross-validation on the training dataset, as proposed by Brouard et al. [45].

In the pre-image step, we assume the MFs of the TMS derivatives of compounds corresponding to the GC-EI-MS spectra from the test dataset to be known: This is certainly true if the GC-EI-MS spectra are generated for testing purposes, as in our case, but note that the MF corresponding to a given MS can be also obtained by using software such as SIRIUS [70]. We use these MFs to generate a candidate set of compounds from PubChem [15] with a MF identical to the MF of each test TMS derivative in turn. The InChIKeY strings of PubChem candidates are retrieved by submitting queries to PubChem’s Power User Gateway through the extensible markup language (XML) and further stored for MFP calculation. For each challenge GC-EI-MS spectrum of a TMS derivative and PubChem candidate, four types of MFPs were calculated (and then concatenated) by using the Chemistry Development Kit (CDK) [71]: substructure fingerprints (307 molecular properties), MACCS fingerprints (166 molecular properties), PubChem (CACTVS) fingerprints (881 molecular properties) and Klekota-Roth fingerprints (4,860 molecular properties), giving 6,214 molecular properties in total. Of these, 3,215 molecular properties were removed, as they were either duplicates or were constant through the entire training dataset. This resulted in 2,999 bit-long vectors describing the structures of the TMS derivatives.

We used IOKR for model learning on both the raw and the curated datasets (CD 0.1 and CD 3.3, respectively; see Fig. 2). We used the learned models to make predictions for the two test datasets, of raw (RAW) and background-subtracted (BS) spectra. All experiments were performed on a computer with a 2.7 GHz Intel Core processor. The computer code was written in Phyton and MATLAB.

## Results and discussion

### Generation of the training dataset

Using the NIST MS Search Software, the initial training dataset of GC-EI-MS spectra (CD 0.1) was generated, consisting of 9,958 GC-EI-MS spectra (Fig. 2). In the first step, the GC-EI-MS spectra of chemically irrelevant compounds were removed. These compounds contained in their chemical structures Si atom(s) that were not part of a TMS group, but belonged to one of the structural categories for exclusion (see "Generation of the training dataset" section). This resulted in the removal of 2,385 GC-EI-MS spectra (24%), yielding the refined dataset (CD 1.1) of 7,573 GC-EI-MS spectra. The remaining collection of GC-EI-MS spectra comprises compounds consisting of the 11 most typical elements in organic chemistry: C, H, N, O, P, S, Br, I, F, Cl and Si [73, 74]. Further, 9 GC-EI-MS spectra of high-mass TMS derivatives (M_w_ > 1000) and 2,925 GC-EI-MS spectra of insufficient quality were removed in the second and third filtration step, respectively. The final training dataset, CD 3.3, consists of 4,648 GC-EI-MS spectra (of 3,948 TMS derivatives), which is 47% of the initial CD 0.1 dataset. After the third filtering step, a final modification in which the *m/z* range was set to *m/z* 50 up to M_w_ + 10 Da was made to the 4,648 spectra remaining in the final version of the training dataset.

### Generation of the test dataset

The predictions, the criteria and the results from the environmental evaluation of the compounds considered for the generation of the test dataset are described in detail in Additional file 2. The evaluation of the 100 compounds selected for generating the test dataset of GC-EI-MS spectra (see Additional file 3) revealed significant environmental relevance for the majority of the test compounds. Briefly, 96 compounds meet at least three RPMBT classification criteria (see "Chemicals and reagents" section), while four compounds (3-methyl-5-nitrocatechol (3M5NC), 4-nitrosyringol (4-NS), 6-hydroxypregnenolone (6HP) and 11-hydroxytestosterone (11HT)) do not, though according to the Regulation (EC) No 1907/2006, Annex XIII [69], they can be considered as persistent, mobile and toxic compounds (Additional file 3).

The derivatization experiments resulted in the formation of 104 TMS derivatives with M_w_ ranges from 182 to 575 Da (see Additional file 4). The optimized derivatization and acquisition conditions can be found in Table 1. During the acquisition, no significant sample contamination or carryover was detected. Baseline subtraction was still performed to remove constantly present background signals, such as those originating from common GC–MS contaminants, e.g., *m/z* 149 as a typical phtalate interference, *m/z* 282, *m/z* 256 and *m/z* 284 for oleic, palmitic and stearic acid, and *m/z* 207, *m/z* 281 and *m/z* 327 of common polysiloxanes resulting from GC column stationary phase degradation. The raw GC-EI-MS spectra were assigned to the RAW test dataset. After background subtraction, the resulting spectra were assigned to the BS test dataset.

### GC-EI-MS spectral similarity analysis and selection

The most widely used, reliable and accurate way of comparing MS spectra is to quantify the fraction of shared peaks by using cosine-based similarity scores that rely on multiplying the intensities of matching peaks [75]. When multiple EI-MS spectra of the same compound are acquired, it is necessary to understand whether each particular MS spectrum should be taken into account, and if not, which one(s) should. To validate the hypothesis that GC-EI-MS spectra of the same compound (here, TMS derivative) are highly reproducible/similar, we performed an all-against-all cosine similarity comparation within the RAW and BS experimental dataset. While the established cosine similarity threshold value is 0.50, the minimum cosine similarity for most of the TMS derivative pairs was higher than 0.95 (Table 2). There are very few TMS derivatives for which a pair of spectra existed either in RAW (2-anilinophenylacetic acid-TMS (2APA-TMS)), BS (cannabidiolic acid TMS (CBDA-TMS), nitroxoline TMS (NX-TMS)) or in both experimental datasets (L-tyrosine TMS (LTYR-TMS), salicylic acid TMS (SA-TMS)), that yielded a minimum cosine similarity factor below 0.50. Despite these few observed discrepancies, we kept all the GC-EI-MS spectra of these TMS derivatives in the experimental datasets.

**Fig. 4:**
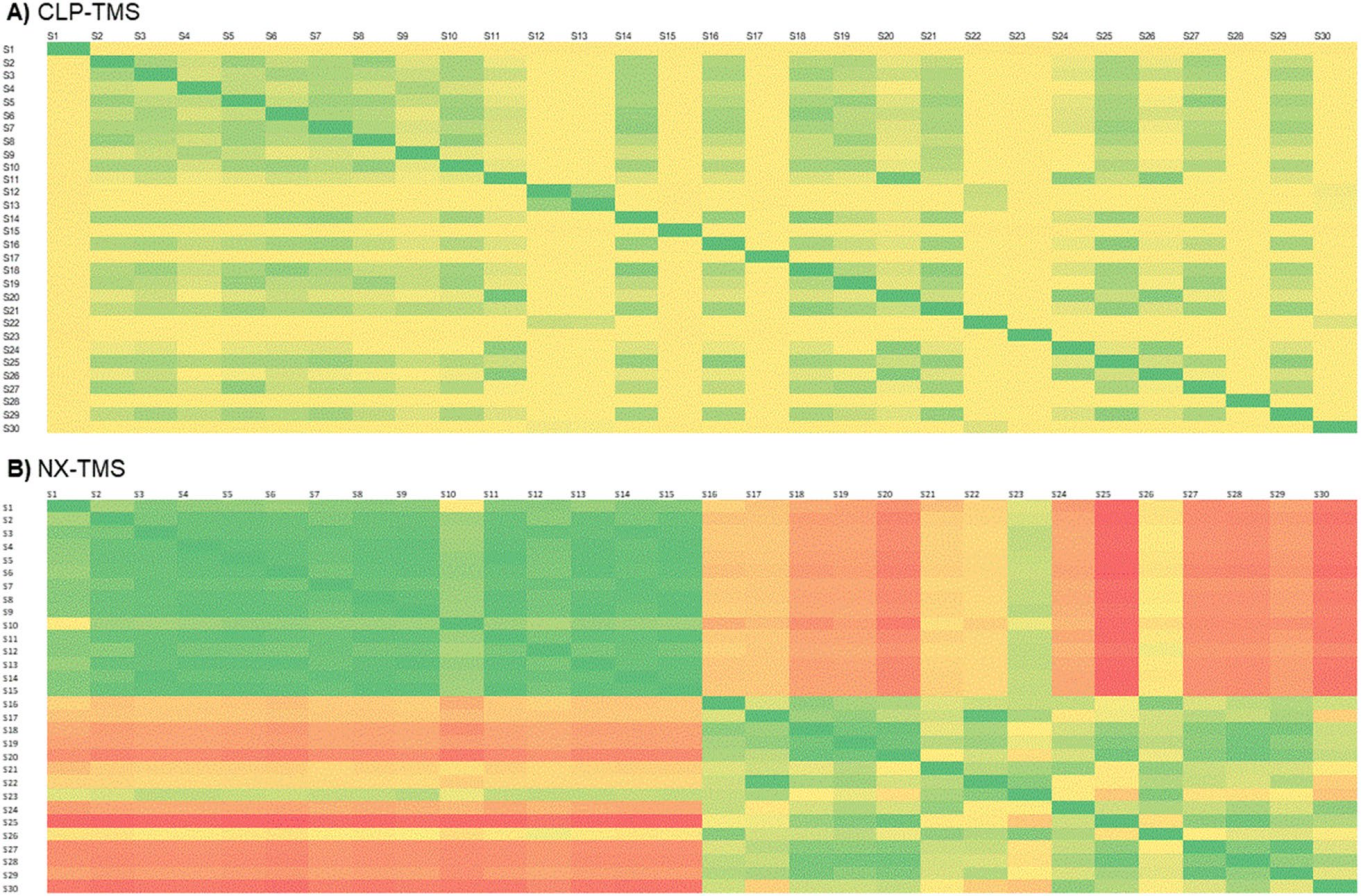
Cosine similarity matrices for multiple spectra of **A:** CLP-TMS RAW and **B:** NX-TMS RAW. Green color indicates high cosine similarity (0.99–1.00), yellow color indicates medium cosine similarity (0.51–0.98) and red color indicates low cosine similarity (below 0.50).

Further, for 2APA-TMS, 17α-ethinyl estradiol TMS (EE2-TMS), estriol TMS (E3-TMS), NX-TMS, LTYR-TMS, L-leucine (LLEU-TMS) and L-serine TMS (LSER-TMS), the minimum cosine similarity between a pair of RAW and BS MS spectra was below 0.50. Moreover, for the latter two TMS derivatives also the maximum cosine similarity factor did not exceed 0.50. Such values indicate that significant changes in MS spectra occur when background subtraction is performed. An example TMS derivative with highly reproducible spectra is given in Fig. 4A, together with an example TMS derivative where GC-EI-MS spectra are less reproducible (Fig. 4B), where green color indicates high cosine similarity (0.99–1.00), yellow color indicates medium cosine similarity (0.51–0.98) and red color indicates low cosine similarity (below 0.50).

Still, the reproducibility of GC-EI-MS spectra of TMS derivatives is overall satisfactory. Any of the acquired GC-EI-MS spectra of each TMS derivative can thus be used to test the CSI:IOKR model. This is clearly visible in Additional file 5, where very few TMS derivatives have pairs of GC-EI-MS spectra of low similarity, i.e., factor below 0.50. Despite these few observed discrepancies, we kept all the GC-EI-MS spectra of these TMS derivatives in the experimental datasets.

### CSI: IOKR

#### The protocol

CSI:IOKR was used to identify CECs from GC-EI-MS spectra of their TMS derivatives. While many different kernels have been proposed in the literature [43–46, 48], it is well known that kernel-based supervised ML methods have computational complexity issues, particularly when using complex kernels. They can have high predictive performance at the price of a heavy computational load. Led by this knowledge, we used two simple kernels, namely the PPK as input and the linear kernel as output kernel. The PPK is computed from a spectrum by modeling each peak in the MS as Gaussian distribution, where the *m/z* ratio and intensity represent the dimensions, and modeling the whole spectrum as a mixture of normal distributions. All-against-all matching is performed by integrating the product between the two corresponding distribution mixtures. This kernel is shown to be superior to simple peak and loss matching kernels computed directly from the spectra (without the knowledge of fragmentation trees) [43, 44]. Among the 24 input kernels of the CSI:IOKR model, PPK was one of the best performing kernels and was assigned the highest weight in the ALIGNF approach of Brouard et al. [45]. The linear kernel was selected as output kernel based on the evaluation results of Brouard et al. [45], where it performed comparably to the polynomial kernel and insignificantly worse than the Gaussian kernel (30.02% vs. 30.66% with the UNIMKL approach, 28.54% vs. 29.78% with the ALIGNF approach). PPK as the input and linear kernel as the output kernel were also the best performing kernels in the IOKRFusion method [47].

The performance of IOKR with the two selected kernels was evaluated on each of the test sets. The identification accuracy was evaluated by using three metrics: (1) the top-k accuracy, that corresponds to the percentage of test TMS derivatives for which the correct structural candidate is found among the top *k* ranked candidates; (2) the average absolute ranking position ($\overline{\mathrm{ARP}}$), the average of ARP values for all CEC-TMS, defined as the number of candidates with better ranking than the correct compound plus 1 and (3) the average relative ranking position ($\overline{\mathrm{RRP}}$), of RRP values for all CEC-TMS [76], calculated as (Eq. 2):

2 $${\text{RRP}} = \frac{1}{2}\left( {1 + \frac{{{\text{BC}} - {\text{WC}}}}{{{\text{TC}} - 1}}} \right)$$

where BC denotes the number of candidates that are better scored than the correct candidate, WC denotes the number of candidates which are ranked lower, i.e., worse than the correct candidate and TC denotes the total number of candidates. The $\overline{\mathrm{RRP}}$ ranges from 0 to 1, with $\overline{\mathrm{RRP}}$ = 0 if the correct candidate is ranked first and $\overline{\mathrm{RRP}}$ = 1 if the correct candidate is ranked last. For each IOKR run, the TMS derivatives missing from the PubChem candidates pool were referred to as “missing”.

#### Performance results

**Fig. 5:**
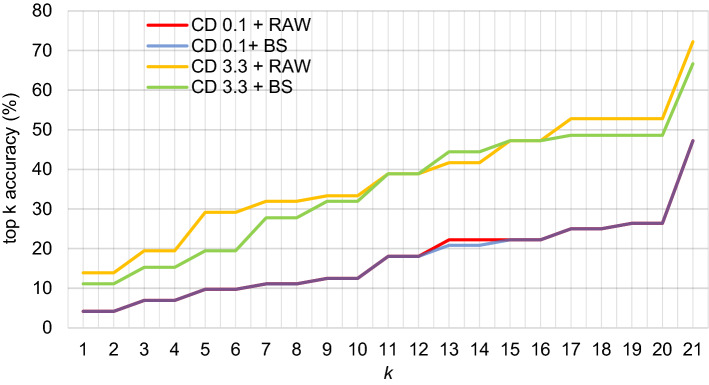
Plot of top-k accuracy for CSI:IOKR with different training and test datasets. CD 0.1 + RAW (red line); CD 0.1 + BS (blue line); CD 3.3 + RAW (yellow line) and CD 3.3 + BS (green line).

The results of evaluating the performance of CSI:IOKR are gathered in Table 2. First, we investigated whether the filtering of the training dataset and the post-acquisition processing of the test dataset affected the performance. The spectral filtering of the training dataset involved the steps illustrated in Fig. 2, whereas the post-acquisition processing only involved baseline subtraction. As evident in Table 2 and Fig. 5, lower performance was achieved when using the unfiltered NIST GC-EI-MS dataset (CD 0.1) in the learning phase, for both test datasets. Two to four-  fold increase of the top-*k* accuracies was observed when the 3-step filtered NIST GC-EI-MS dataset (CD 3.3) was used to train the model (instead of CD 0.1). Also, the $\overline{\mathrm{ARP}}$ and $\overline{\mathrm{RRP}}$ improved two-fold with the CD 3.3 dataset. For example, the $\overline{\mathrm{ARP}}$ of the correct TMS derivative was 31 positions and 29 positions higher for the RAW and BS datasets, respectively. As evident in Fig. 5, very subtle differences of less than 2% appeared between performance on the RAW and BS test datasets in all experiments, slightly favoring the RAW test dataset, especially when CD 3.3 was used to train the model. However, the $\overline{\mathrm{RRP}}$ values were comparable for both the RAW and the BS test sets with both the CD 0.1 and the CD 3.3 training sets, confirming that this baseline subtraction is not important for the identification task. Therefore, we consider that the CSI:IOKR model performs best when trained using the CD 3.3 training dataset and tested on RAW test dataset. Thus, further evaluation of the CSI:IOKR performance is done based on the results from CD 3.3 + RAW.

**Table 2:** The identification accuracies of CSI:IOKR on different training and test datasets.

Training dataset	Test dataset	Presence of the test compounds in training dataset	Number of test compounds	Missing	Top 1	Top 10	Top 20	$\overline{\mathrm{ARP}}$	$\overline{\mathrm{RRP}}$
				n (%)	n (%)	n (%)	n (%)		
CD 0.1	RAW	Yes	63	9 (14.3)	1 (1.6)	10 (15.9)	18 (28.6)	59.8	0.79
CD 0.1	RAW	No	41	23 (56.1)	2 (4.9)	9 (22.0)	16 (39.0)	24.7	0.69
CD 0.1	RAW	Merged	104	32 (31.8)	3 (2.9)	19 (18.3)	34 (32.7)	52.0	0.77
CD 0.1	BS	Yes	62	8 (12.9)	1 (1.6)	10 (16.1)	18 (29.0)	60.0	0.79
CD 0.1	BS	No	41	23 (56.0)	2 (4.9)	9 (22.0)	16 (39.0)	24.9	0.72
CD 0.1	BS	Merged	103	31 (30.1)	3 (2.9)	19 (18.5)	34 (33.0)	52.2	0.77
CD 3.3	RAW	Yes	63	9 (14.3)	7 (11.1)	25 (39.7)	37 (58.7)	23.8	0.37
CD 3.3	RAW	No	41	23 (56.1)	4 (9.8)	14 (34.2)	16 (39.0)	11.3	0.35
CD 3.3	RAW	Merged	104	32 (30.8)	11 (10.6)	39 (37.5)	53 (51.0)	21.0	0.36
CD 3.3	BS	Yes	62	8 (12.9)	4 (6.5)	24 (38.7)	36 (58.1)	26.2	0.39
CD 3.3	BS	No	41	23 (56.1)	5 (12.2)	14 (34.2 5)	16 (39.0)	11.0	0.36
CD 3.3	BS	Merged	103	31 (30.1)	9 (8.7)	38 (36.9)	52 (50.5)	22.8	0.38

For each experimental setup, the total number of CEC-TMS derivatives, the number (n) and percentage (%) of missing CEC-TMS derivatives, and CEC-TMS derivatives correctly ranked in the top 1, 10 and 20 hits (top k accuracies), average absolute ranking position ($\overline{\mathrm{ARP}})$ and average relative ranking position ($\overline{\mathrm{RRP}})$ are given.

For each experimental setup, the total number of CEC-TMS derivatives, the number (n) and percentage (%) of missing CEC-TMS derivatives, and CEC-TMS derivatives correctly ranked in the top 1, 10 and 20 hits (top k accuracies), $\overline{\mathrm{ARP}}$ and $\overline{\mathrm{RRP}}$ are given.

Further, we compared the performance of CSI:IOKR for two subgroups of TMS derivatives from the test set, i.e., those with GC-EI-MS spectra within and outside the training dataset (»presence in training dataset« Yes/No, Table 2). The results show better identification performance for the GC-EI-MS spectra that were part of the training dataset for the CD 3.3 dataset. The differences in performance are small and their direction is unclear for the CD 0.1 training dataset, expecially for the top 1 metric. The underlying reason may be that the size of the candidate sets was typically much lower for the group of TMS derivatives that were not part of the training dataset, reflecting the high number of TMS derivatives that are not part of PubChem.

**Fig. 6:**
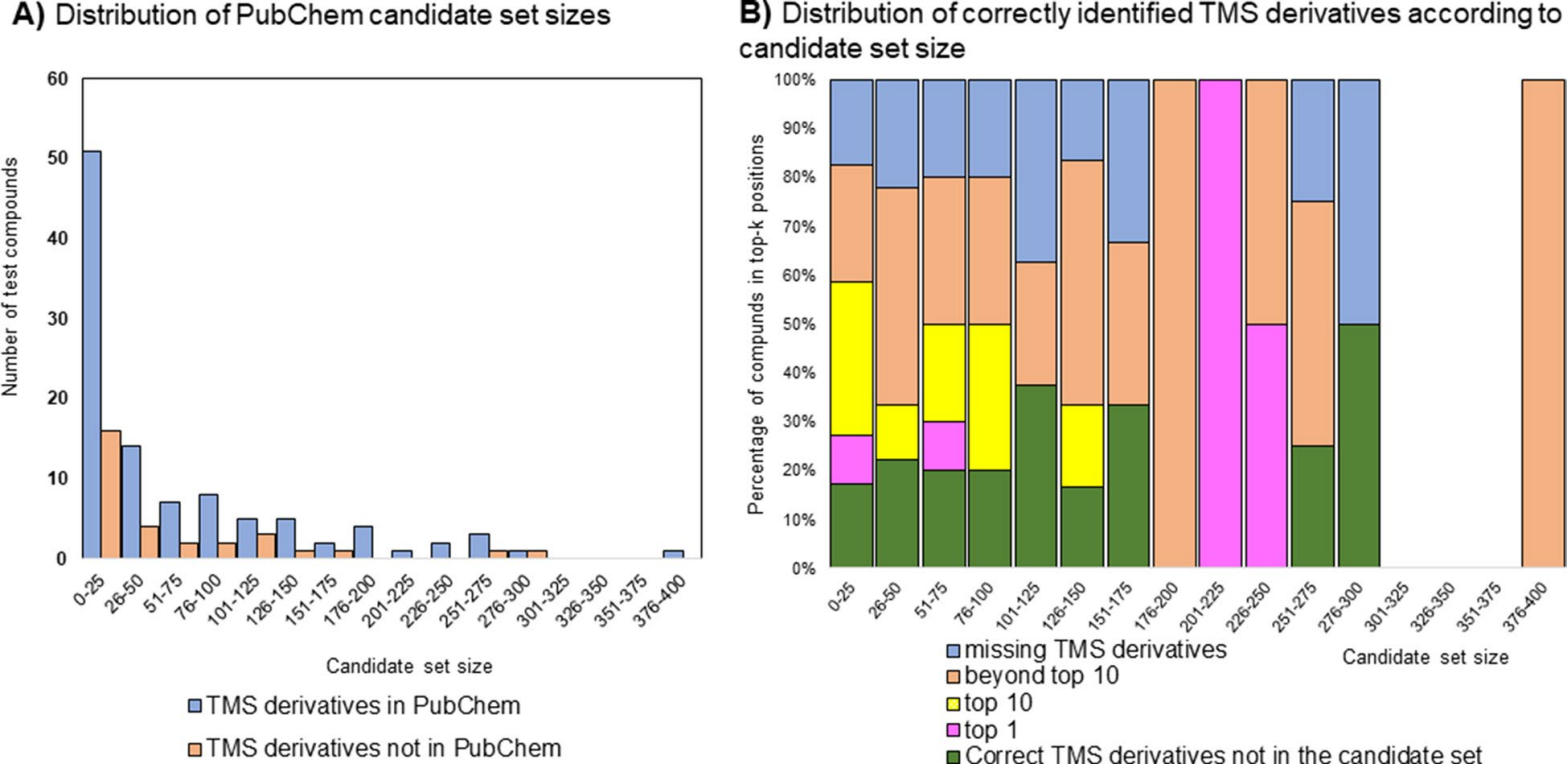
**A**: Distribution of PubChem candidate set sizes for the test set spectra. Blue color represents the TMS derivatives that are in PubChem, and orange color represents the TMS derivatives that are not in PubChem; **B**: Distribution of TMS derivatives from test dataset; the y-axis represents the percentage of correct TMS derivatives not present in their candidate set (green color); percentage of correct TMS derivatives that are ranked top 1 (pink color); percentage of correct TMS derivatives ranked in the top 10 (yellow color); percentage of TMS derivatives that are ranked below the top 10 positions (orange color) and percentage of missing TMS derivatives, i.e., TMS derivatives that are not in the PubChem candidate set (blue color) among the test TMS derivatives with candidate set sizes in each size bin, while the x-axis represents the candidate set size bins.

With this in mind, we investigated the relation between candidate set size and identification performance. The distribution of candidate sets sizes is presented in Fig. 6. The maximum size of a candidate set was less than 400, while the majority candidate sets (about 50%) consisted of 0–25 candidates (Fig. 6A). According to the results (Fig. 6B), the difficulty of the identification task does not seem to strongly depend on the size of the candidate set, as the method is able to correctly identify a significant proportion of test compounds within the top 1 and top 10 candidates, even for larger candidate sets [45]. For 32 challenges from the test dataset, their corresponding candidate sets did not contain the correct compound.

**Fig. 7:**
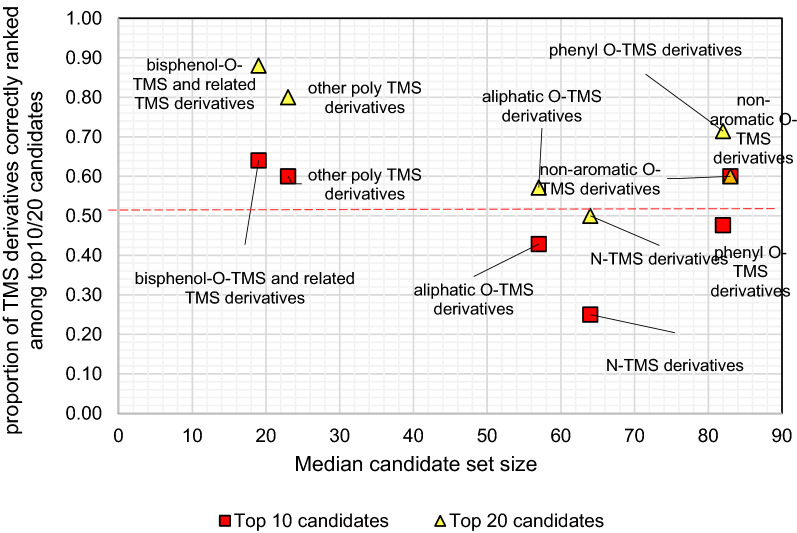
The percentage of candidates ranked higher than the correct compound versus the size of each PubChem candidate set. Red triangles represent the percentage of TMS derivatives that are not present in the training dataset; the yellow squares represent the percentage of TMS derivatives that are present in the training dataset.

Relating the number of candidates within each PubChem candidate set with the percentage of candidates ranked higher than the correct compound (Fig. 7) did not reveal any specific pattern, regardless of whether the TMS derivatives had their spectra within or outside of the training dataset. The results indicate that the influence of the size of the PubChem candidate sets on the identification accuracy is negligible. That is, the CSI:IOKR model, in a percentage-wise manner, does not perform worse with larger candidate sets. However, this may not yield satisfactory performance when the correct compound is, for example, ranked at position 100 among 1000 candidates. In this case, the percentage is good, while the rank itself is not.

**Fig. 8:**
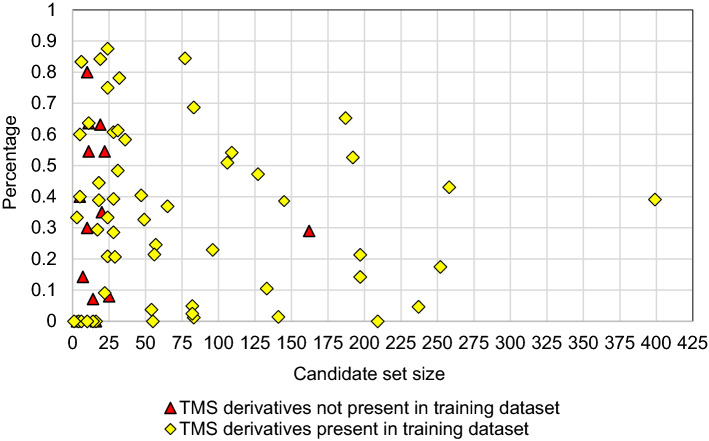
The median number of candidates in the candidate sets of different classes of TMS derivatives plotted against the proportion of challenge TMS derivatives within the clusters correctly ranked within the top 10 candidates (red squares) and top 20 candidates (yellow triangles).

**Fig. 9:**
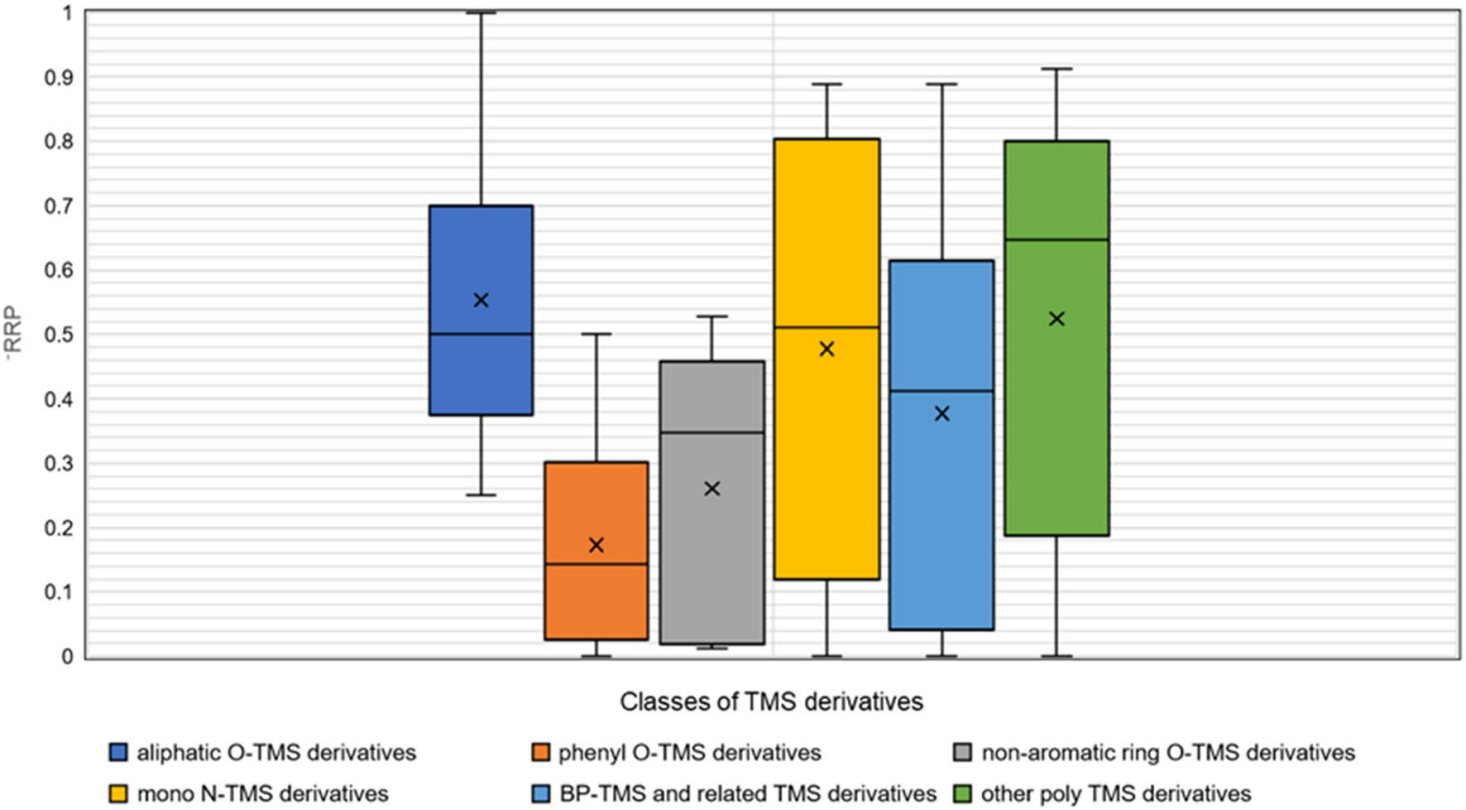
Box plot representing the distribution of the $\overline{\mathrm{RRP}}$ of the challenge compounds in each class of TMS derivatives. Dark blue plot represents the aliphatic O-TMS derivatives, orange plot represents phenyl O-TMS derivatives, grey plot represents the non-aromatic ring O-TMS derivatives, the yellow plot represents the mono N-TMS derivatives, the light blue plot represents the bisphenol-TMS and related TMS derivatives, while the green plot represents the other poly TMS derivatives.

In order to investigate the ability of CSI:IOKR to identify particular groups of TMS derivatives, we divided the latter into 6 structural TMS classes, based on the moiety that the TMS group was attached to (Additional file 6). For each TMS class, the median number of candidates in all candidate sets in the class was plotted against the proportion of TMS derivatives for which the correct candidate was ranked among top 10 and top 20 candidates (Fig. 8) and average $\overline{\mathrm{RRP}}$ (Fig. 9). The TMS derivatives for which the correct candidate was absent from the corresponding candidate sets were omitted.

For all TMS classes, CSI:IOKR performs satisfactorily both in terms of the proportion of TMS derivatives correctly ranked among the top10/20 candidates and in terms of the $\overline{\mathrm{RRP}}$ of the challenge TMS derivatives. Except for aliphatic O-TMS derivatives and N-TMS derivatives,  ≥ 50% of the correct TMS derivatives are ranked among the top 10 candidates. Especially good ranking scores are achieved for the poly TMS derivatives, i.e., bisphenol O-TMS derivatives and related TMS derivatives, and the other poly TMS derivatives, including mixed N, O-TMS and N-TMS derivatives, that have highest M_w_ and lowest median candidate size, which may partially contribute to their relatively good ranking. Namely, the correct CEC-TMS was ranked on average positions 10.68 and 19.50, respectively, while the average PubChem candidate set size was 22.04 and 28.60, respectively, which is 2–5 times lower than the values for the other TMS classes. Also evident from Fig. 8 is that CSI:IOKR performs solidly for phenyl O-TMS and nonaromatic O-TMS derivatives, which yield relatively high average candidate set sizes (108.43 and 120.67, respectively, data not shown). Despite that, their ranking scores are satisfactory, as well as their average $\mathrm{RRP}$. The class of non-aromatic O-TMS derivatives contains 5 CEC-TMS derivatives, and thus the number of CEC-TMS derivatives is not representative, so that solid conclusions can be extracted. On the other hand, the phenyl O-TMS class is represented by 21 CEC-TMS, with low average ranking position (19.14), but high average PubChem candidate set size (108.43). Here, a factor that may positively contribute to the good ranking of some structural classes is the specificity of the fragmentation patterns, leading to uniqueness of its GC-EI-MS spectrum, which is responsible for the good ranking, independent of the size of the PubChem candidate set. Finally, $\overline{\mathrm{RRP}}$ is  > 0.50 or close to 0.50 (the threshold of satisfactory accuracy) for all TMS classes, except for phenyl-O-TMS derivatives (data not shown).

**Fig. 10:**
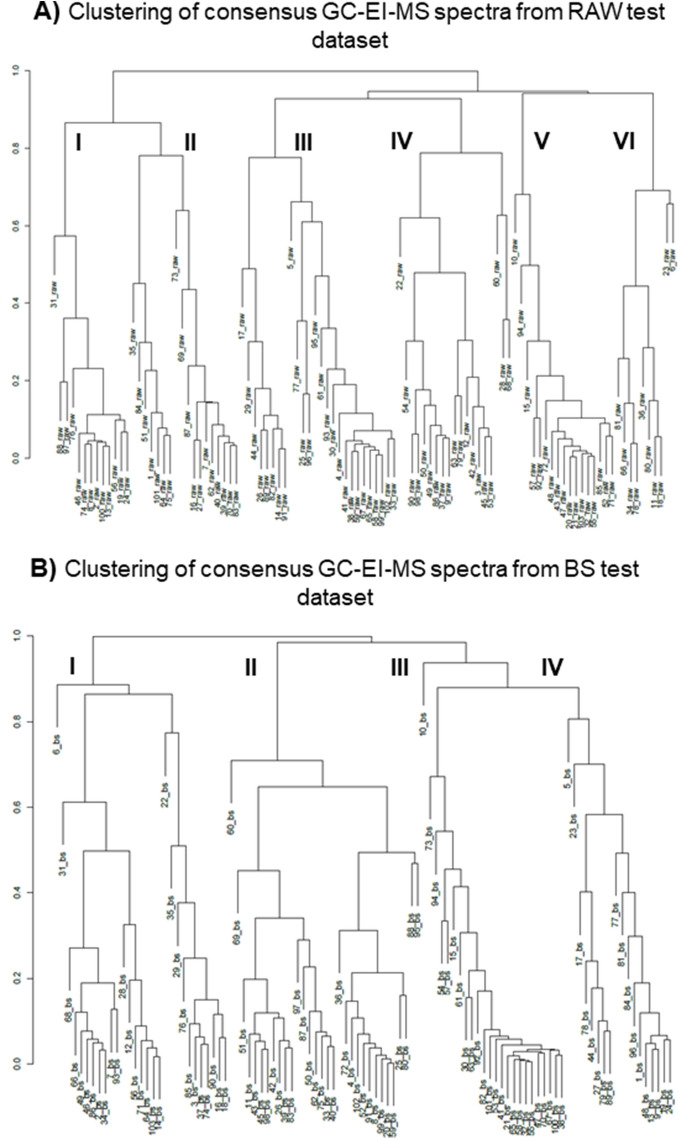
Clustering of **A:** consensus GC-EI-MS spectra from RAW test dataset and **B:** consensus GC-EI-MS spectra from BS test dataset based on similarity of MS behavior and properties.

**Fig. 11:**
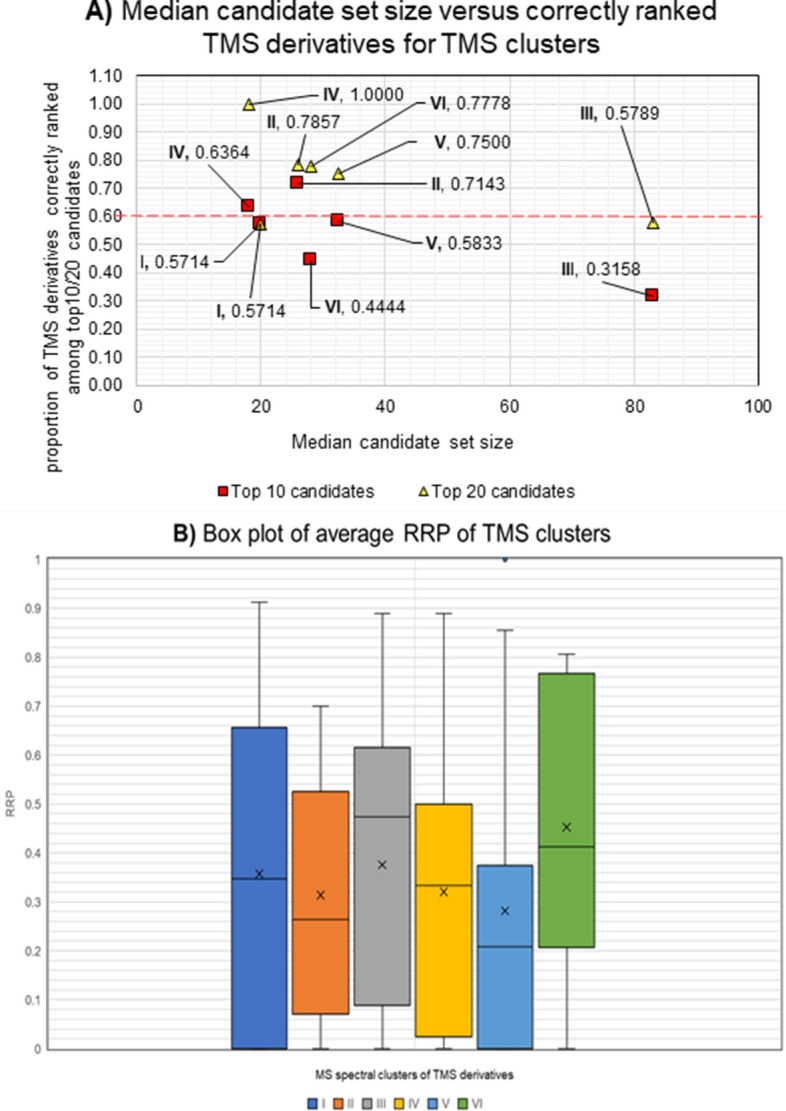
**A** The median number of candidates in the candidate sets of different clusters of TMS derivatives (RAW dataset) plotted against the proportion of challenge TMS derivatives within the cluster correctly ranked within the top 10 candidates (red squares) and top 20 candidates (yellow triangles). **B** Box plot representing the different $\overline{\mathrm{RRP}}$ values for the challenge compounds in each cluster of TMS derivatives from RAW dataset (bottom): dark blue plot—I, orange plot—II, grey plot—III, yellow plot—IV, light blue plot—V and green plot—VI.

Legend: 1: BPAF-2TMS; 2: DH-BP-2TMS; 3: 2APA-TMS; 4: 3M5NC-2TMS; 5: CLP-TMS; 6: 3MC-2TMS; 7: 4,4'-BP-2TMS; 8: HPP-TMS; 9: H-BP-TMS; 10: 4NC-2TMS; 11: 4NG-TMS; 12: 4NS-TMS; 13: 4NP-TMS; 14: 4OP-TMS; 15: 5AD-TMS; 16: 5NG-TMS; 17: 6HP-TMS; 18: 6MAM-TMS; 19: 6NG-TMS; 20: 8HQ-TMS; 21: 9HF-TMS; 22: 11HAD-TMS; 23: 11HT-2TMS; 24: 11-OH-THC-2TMS; 25: 11N9THC-2TMS; 26: E2-2TMS; 27: EE2-TMS; 28: 17HP-TMS; 29: AA-2TMS; 30: AMP-TMS; 31: PAA-TMS; 32: BA-TMS; 33: BZECG-TMS; 34: BzPb-TMS; 35: 22BPF-2TMS; 36: 24BPF-2TMS; 37: BPA-2TMS; 38: BPAP-2TMS; 39: BPB-2TMS; 40: BPBP-2TMS; 41: BPC-2TMS; 42: BPCL-2TMS; 43: BPE-2TMS; 44: BPF-2TMS; 45: BPFL-2TMS; 46: BPM-2TMS; 47: BPP-2TMS; 48: BPPH-2TMS; 49: BPS-2TMS; 50: BPZ-2TMS; 51: BD-TMS; 52: BP26DM-2TMS; 53: BuPb-TMS; 54: BHT-TMS; 55: CBC-TMS; 56: CBD-2TMS; 57: CBDA-3TMS; 58: CBN-TMS; 59: CBZ-TMS; 60: CAT-2TMS; 61: CA-4TMS; 62: CLA-TMS; 63: COD-TMS; 64: THC-TMS; 65: THCA-2TMS; 66: DF-TMS; 67: BP-8-2TMS; 68: ERY-4TMS; 69: E3-3TMS; 70: E1-TMS; 71: EtPb-TMS; 72: ET-TMS; 73: IB-TMS; 74: IbUPb-TMS; 75: IPrPb-TMS; 76: LLEU-TMS; 77: LAA-4TMS; 78: LLEU-2TMS; 79: LSER-3TMS; 80: LTYR-3TMS; 81: MCA-2TMS; 82: MAMP-TMS; 83: MePb-TMS; 84: MORPH-2TMS; 85: NAP-TMS; 86: NX-TMS; 87: OCA-2TMS; 88: PCA-2TMS; 89: PrPb-TMS; 90: QA-5TMS; 91: RES-2TMS; 92: SA-2TMS; 93: SA-TMS; 94: SHA-4TMS; 95: STA-2TMS; 96: STA-TMS; 97: SFA-2TMS; 98: SFA-TMS; 99: SYR-TMS; 100: T3HC-TMS; 101: TCS-TMS; 102: DHDPE-2TMS; 103: UA-2TMS.

Clustering of MS spectra for the RAW (Fig. 10A) and the BS dataset (Fig. 10B) revealed 6 and 4 clusters, respectively. The RRP and proportion of TMS derivatives ranked among top 10/20 candidates differed significantly between the clusters of TMS derivatives with significant MS spectral similarity. The median candidate sizes for all clusters (except for cluster 3) were  < 35 candidates. For all of them (except for cluster 6, where the top 10 ratio is 0.44), top 10 and top 20 ratios of  > 0.55 were achieved (Fig. 11A). $\overline{\mathrm{RRP}}$ values vary significantly within all clusters, with average $\overline{\mathrm{RRP}}$ <0.60 and clusters 2 and 5 having the lowest average $\overline{\mathrm{RRP}}$s (0.26 and 0.21) (Fig. 11B).

Overall, the performance of the CSI:IOKR model for identification of TMS derivatives using GC-EI-MS spectra is somewhat lower as compared to its performance on a benchmark dataset, represented by 4,138 LC–ESI–MS/MS spectra from the Global Natural Products Social (GNPS) library [45]. This might be due to the smaller size of our test dataset or the type of input data (LC–ESI–MS/MS vs. GC-EI-MS). Interestingly, CSI:IOKR in our study resulted in identical median ARP as MetExpert for TMS derivatives, with slightly lower top 1 (11% vs. 13%) and remarkably better top 15 accuracy (63% vs. 52%).

## Conclusions and further perspectives

The rate, volume and variety of compounds being introduced to the environment continues to expand exponentially. Consequently, many research groups and regulatory agencies are developing computational and high-throughput approaches for CEC annotation. As ML-based approaches are the future of CEC annotation, exploiting the perspectives for their further use is of utmost importance. Here we show that ML approaches, which have been predominantly used to annotate CEC from LC–MS data, can also be used to address the task of annotating TMS derivatives of CECs from GC–MS data. More specifically, this study shows that CSI:IOKR can be successfully employed for the annotation of TMS derivatives of CEC from GC-EI-MS data. This presents a viable alternative to MSL search independent of an instrumental platform and data processing software.

Importantly, this study shows that expert curation of spectral datasets crucially improves the identification performance of ML-based approaches. Furthermore, CSI:IOKR is useful in the identification of CEC that have been previously characterized (i.e., known unknowns that are currently in compound DBs) but whose GC-EI-MS spectra are not included in MSLs, thus increasing our knowledge on the composition of environmental samples. While spectral comparisons with reference standards or de novo structural elucidations might be required to validate the predictions, CSI:IOKR provides an efficient approach to prioritize candidates and reduces the time spent for compound annotation.

As further work, we propose a few straightforward extensions of this research that could be potentially successful and useful in enhancing the employment of the CSI:IOKR method in GC–MS-based CECs annotation. Instead of the PubChem repository, middle-sized compound DBs of particular value to the environmental science and toxicology communities, such as the US EPA’s CCD [11], can be used. These compound DBs were proven to have higher potential in compound structure identification and exposure risk assessment over large repositories, such as ChemSpider [16] and PubChem [15, 17]. Moreover, the potential of CSI:IOKR could be further exploited on GC-EI-MS spectral data of TBDMS derivatives.

However, the ultimate challenge for IOKR would be the identification of the underivatized (parent) compounds using the GC-EI-MS spectra of their silyl derivatives. The employment of IOKR and other IOKR-based methods would be significantly encouraged by their implementation within existing and upcoming CA frameworks. Besides CSI:IOKR, it would be very beneficial if other IOKR approaches [46, 47] and other cutting-edge ML-based methods [48, 49] are also challenged against identifying CECs using GC-EI-MS spectra. In that spirit, we would like to encourage the use of the generated GC-EI-MS datasets as benchmark datasets for further evaluation and improvement of ML-based approaches in GC–MS-based compound annotation.

## Supplementary Information

**Additional file 1. **Specifications and physico-chemical properties of the selected CECs and their TMS derivatives for the test dataset.

**Additional file 2. **Environmental relevance evaluation of the selected compounds according to the RPMBT system.

**Additional file 3. **Representation of the environmental relevance evaluation**.**

**Additional file 4. ** Chemical structures, IUPAC names, common names and abbreviations, CAS numbers and molecular weights of the CECs and their TMS derivatives from the test dataset.

**Additional file 5. ** Cosine similarity values for pairs of GC-EI-MS spectra for each TMS derivative.

**Additional file 6. ** Structural classification of CEC-TMS derivatives.

## Data Availability

The source code of CSI:IOKR is available on GitHub at https://github.com/aalto-ics-kepaco/PPIprediction-w-IOKR-MKL. The training data were curated from the commercially available NIST Mass Spectral Library 17. Due to NIST’s individual license, restricting the use to a single computer that is not accessible by more than one person, the training data cannot be made available to the public by the authors. However, with licensed access to the NIST 17 MSL, the training data can be reconstructed following the detailed description of data preparation given in Sect. 3.1. All testing datasets and accompanying metadata were created by the authors and can be accessed at and downloaded entirely or partially in several formats (.txt, .xlsx) from the following link: https://data.mendeley.com/datasets/j3z5bmvmnd/3.

## Abbreviations

11HT: 11-hydroxytestosterone
2-APA: 2-anilinophenylacetic acid
3M5NC: 3-methyl-5-nitrocatechol
4NS: 4-nitrosyringol
6HP: 6-hydroxypregnenolone
$\overline{\mathrm{ARP}}$: Average absolute ranking position
BS: Background subtraction
CASMI: Critical Assessment of Small Molecule Identification
CBDA: cannabidiolic acid
CCD: Comptox Chemistry Dashboard
CEC: Contaminant of emerging concern
CSI: Compound structure identification
CSI:IOKR: Compound Structure Identification:Input Output Kernel Regression
DB: Database
DSSTox: Distributed Structure-Searchable Toxicity
E3: estriol
EC: European Commission
EE2: 17α-ethinyl estradiol
EI: Electron impact
EPI: Estimation Programs Interface
ESI: Electrospray ionization
GC: Gas chromatography
GMD: Golm Metabolome Database
GNPS: Global Natural Products Social
HR/AM-MS: High resolution/accurate mass—mass spectrometry
IOKR: Input Output Kernel Regression
LC: Liquid chromatography
LLEU: L-leucine
LOEC: Lowest observed effect concentration
LSER: L-serine
LTYR: L-tyrsoine
MF: Molecular formula
MFP: Molecular fingerprint
ML: Machine learning
MS: Mass spectrometry
MS/MS: Tandem mass spectrometry
MSL: Mass spectral library
M_w_: Molecular weight
NIST: National Institute of Standards and Technology
NOEC: No observed effect concentration
NX: nitroxoline
*m/z*: Mass-to-charge ratio
PPK: Product kernel
REACH: Registration, Evaluation, Authorisation and Restriction of Chemicals
$\overline{\mathrm{RRP}}$: Average relative ranking position
SA: salicylic acid
SSS: Stock standard solution
SVM: Support vector machine
T3DB: Toxin and-Toxin Target database
TBDMS: *tert*-butyl dimethylsilyl
T.E.S.T: Toxicity Estimation Software Tool
TMCS: Trimethylchlorosilane
TMS: Trimethylsilyl
WS: Working solution
XML: Extensible markup language
