# Supplementary material for: Machine learning for identification of silylated derivatives from mass spectra
Source: J Cheminform. 2022 Sep 15;14:62. doi: 10.1186/s13321-022-00636-1 (PMC9476372; doi:10.1186/s13321-022-00636-1)
Supplement: Supplementary file 1 — Additional file 1. Specifications and physico-chemical properties of the selected CECs and their TMS derivatives for the test dataset. [file 13321_2022_636_MOESM1_ESM.docx]

**Machine learning for identification of silylated derivatives from mass spectra**

Milka Ljoncheva ^†, ‡^, Tomaž Stepišnik ^∫, ‡^, Tina Kosjek ^†, ‡^, Sašo Džeroski ^∫, ‡, *^

*^†^ Jozef Stefan Institute, Department of Environmental Sciences, Jamova 39, 1000 Ljubljana, Slovenia*

*^∫^ Jozef Stefan Institute, Department of Knowledge Technologies, Jamova 39, 1000 Ljubljana, Slovenia*

*^‡^ Jozef Stefan International Postgraduate School, Jamova 39, 1000 Ljubljana, Slovenia*

**Additional file 1**

**Specifications and physico-chemical properties of the selected CECs and their TMS derivatives for the test dataset**

Analytical standards of bisphenol A (BPA; > 97%) was purchased from Merck (Darmstadt, Germany). Analytical standards of benzoic acid (BA; 99%), 2,2′-methylenediphenol (22BPF; 98%), 4,4′-biphenol (4,4'-BP; 98%), 4,4'-dihydroxydiphenyl ether (DHDPE; 98%), 4,4′-isopropylidenebis(2,6-dimethylphenol) (BP26DM; 98%), 2,4'-methanediyldiphenol (24BPF; >98%), bisphenol AF (BPAF; > 99%), bisphenol AP (BPAP; > 99%), bisphenol C (BPC; > 99%), bisphenol E (BPE; > 98%), bisphenol F (BPF; > 98%), bisphenol FL (BPFL; > 99%), bisphenol M (BPM; 99%), bisphenol BP (BPBP; > 98%), bisphenol P (BPP; > 99%), bisphenol S (BPS; > 98%), bisphenol Z (BPZ; > 98%), 2-benzyl-4-chlorophenol (clorophene, CLP; 95%), citric acid monohydrate (CA; ≥99.9%), 4-cumylphenol (HPP; > 99%), 2,4-dihydroxybenzophenone (DH-BP; > 99%), estrone (E1; 99%), 17β-estradiol (E2; 98%), estriol (E3; ≥ 97%), 17α-ethynyl estradiol (EE2; 98%), 4-hydroxybenzophenone (H-BP; > 97%), 2,2'-dihydroxy-4-methoxybenzophenone (BP-8; > 97%), clofibric acid (CLA; 97%), ibuprofen (IB; > 98%), naproxen (NAP; 98%), ketoprofen (KET; > 98%), diclofenac (DF; 97%), methylparaben (MePb; 98.5%), ethylparaben (EtPb; 99%), propylparaben (PrPb; 99%), butylparaben (BuPb; 99%), isobutylparaben (iBuPb; 99%), benzylparaben (BzPb; 99%), meso-erythritol (ERY; ≥99.0%), 4-nonylphenol (4-NP; 97.0%), phenylacetic acid (PAA; ≥ 99.0%), (-)-quinic acid (QA; 98.0%), resorcinol (RES; 99.0%), salicylic acid (SA, ≥ 99.0%), shikimic acid (SHA; ≥ 99.0%), urea (UA; 98.0%), sulfanilamide (SFA; ≥99%), adipic acid (AA; 96%), 4-tert-octylphenol (4-OP; 97.0%), 9-hydroxyfluorene (9-HF; 96%), butylated hydroxytoluene (BHT; ≥ 99%), L-leucine (LLEU; ≥98%), L-serine (LSER; ≥ 99%), L-tyrosine (LTYR; ≥ 98%), L-ascorbic acid (LAA; 99.0%), m-coumaric acid (MCA; ≥95.0%), p-coumaric acid (PCA; ≥ 98.0%), o-coumaric acid (OCA; 97.0%) and triclosan (TCS; 97%) were purchased from Sigma Aldrich (Steinheim, Germany). Analytical standards of (+)-cannabidiol (CB; 1 mg/mL in methanol (MeOH); 99.8%), cannabinol (CBN; 1 mg/mL in MeOH; 99.6%), cannabichromene (CBC; 1 mg/mL in MeOH; 99.2%), cannabidiolic acid (CBDA; 1 mg/mL in acetonitrile (ACN)), (-)-Δ^9^ tetrahydrocannabinol (Δ^9^-THC; 1 mg/mL in MeOH; 98.1%), (-)-Δ^9^ tetrahydrocannabinolic acid (Δ^9^-THCA; 1 mg/mL in ACN; 98.9%), trans-3’-hydroxycotinine (T3HC; 1 mg/mL in MeOH; 99.5%), benzoylecgonine (BZECG; 1 mg/mL in MeOH; 99.9%), codeine (COD; 1 mg/mL in MeOH; 99.8%), morphine (MORPH; 1 mg/mL in MeOH; 99.4%), 6-monoacetylmorphine (6-MAM; 100 µg/mL in ACN; 99.4%), (±)-11-hydroxy-Δ^9^-tetrahydrocannabinol (11-OH-THC; 1 mg/mL in MeOH; 99.5%), (±)-11-nor-9-carboxy-Δ9-tetrahydrocannabinol (11N9CTHCA; 1 mg/mL in MeOH, >99.9%), (±)-amphetamine (AMP; 1 mg/mL in MeOH) and (±)-methamphetamine (MAMP; 1 mg/mL in MeOH) were purchased from Cerilliant (Darmstadt, Germany). Carbamazepine (CBZ; 99%) was purchased from Acros Organics (New Jersey, USA), isopropyl paraben (iPrPb; 99.2 %) from Carbone Scientific (London, UK) and bisphenol B (BPB; > 99%) from Dr. Ehrenstorfer GmbH (Augsburg, Germany). Bisphenol Cl (BPCL; 98 %) and bisphenol PH (BPPH; 98%) were purchased from ABCR GmbH (Karlsruhe, Germany). 8-hydroquinoline (8-HQ; 99%) was purchased from Reidel de Haën (Seelze, Germany). 2-anilinophenylacetic acid (2APA) was custom synthesized at the Faculty of Chemistry and Chemical Technology, University of Ljubljana (Ljubljana, Slovenia). 4-nitroguaiacol (4NG), 5-nitroguaiacol (5NG), 6-nitroguaiacol (6NG), catechol (CAT), 3-methylcatechol (3MC), 3-mehyl-5-nitrocatechol (3M5NC), 4-nitrocatechol (4NC), syringol (SYR) and 4-nitrosyringol (4-NS) were custom synthesized at the Faculty of Medicine, University of Ljubljana (Ljubljana, Slovenia). Etofylline (ET) and nitroxoline (NX) were shared reference standards in the frame of the HMB4E project. 17α-hydroxyprogesterone (17HP), 6β-hydroxypregnenolone (6HP), 5-androsten-3β, 17β-diol (5AD), 5α-dihydrotestosterone (boldenone, BD), 11α-hydroxytestosterone (11HT), 11α-hydroxyandrostenedione (11HAD) and dihydrotestosterone (stanolone, ST) were custom synthesized.

The derivatization agents N-methyl-N-(trimethylsilyl) trifluoroacetamide (MSTFA), N, O-bis(trimethylsilyl)trifluoroacetamide (BSTFA) and N, O-bis(trimethylsilyl)trifluoroacetamide with 1% trimethylchlorosilane (BSTFA + 1% TMCS) were purchased from Sigma-Aldrich (Sigma-Aldrich Laborchemikalien GmbH, Steinheim, Germany). ACN, ethylacetate (EtAc) and MeOH were purchased from JT Baker (Deventer, the Netherlands). All chemicals are with analytical grade purity.
