# Supplementary material for: Machine learning for identification of silylated derivatives from mass spectra
Source: J Cheminform. 2022 Sep 15;14:62. doi: 10.1186/s13321-022-00636-1 (PMC9476372; doi:10.1186/s13321-022-00636-1)

**Machine learning for identification of silylated derivatives from mass spectra**

Milka Ljoncheva ^†, ‡^, Tomaž Stepišnik ^∫, ‡^, Tina Kosjek ^†, ‡^, Sašo Džeroski ^∫, ‡, *^

*^†^ Jozef Stefan Institute, Department of Environmental Sciences, Jamova 39, 1000 Ljubljana, Slovenia*

*^∫^ Jozef Stefan Institute, Department of Knowledge Technologies, Jamova 39, 1000 Ljubljana, Slovenia*

*^‡^ Jozef Stefan International Postgraduate School, Jamova 39, 1000 Ljubljana, Slovenia*

**Additional file 3**

**Representation of the environmental relevance evaluation**

Environmental relevance evaluation according to the RPMBT system. Green fields with thick marks (✓) indicate fulfillment of the criterium, yellow fields with ✓ marks indicate conditional fullfillment of criterium; blue fields with ✓ marks indicate compounds that do not fulfill the criteria, but according to the European Commission Regulation (EC) No.1907/2006 of the European Parliament and of the Council on the Registration, Evaluation, Authorisation and Restriction of Chemicals (REACH) can be considered as Persistent, Mobile and Toxic compounds; red fileds with (🗶) marks indicate not fullfilled criterium.
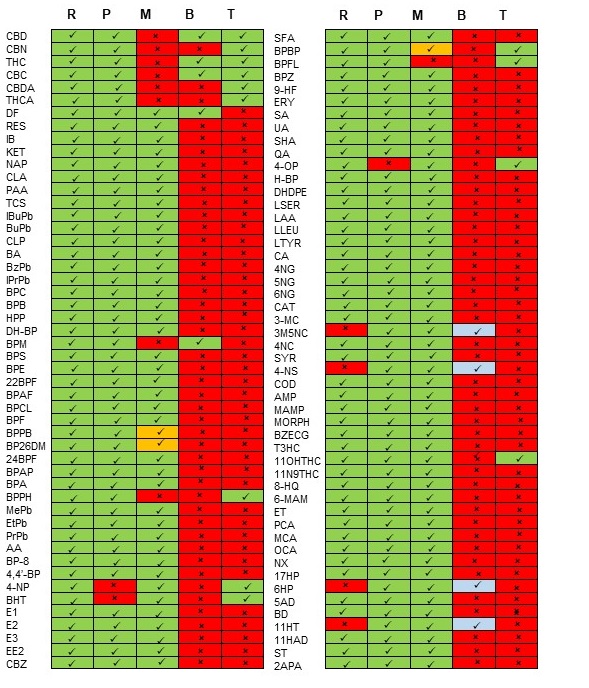

Supplement: Supplementary file 3 — Additional file 3. Representation of the environmental relevance evaluation. [file 13321_2022_636_MOESM3_ESM.docx]
