# Supplementary material for: Machine learning for identification of silylated derivatives from mass spectra
Source: J Cheminform. 2022 Sep 15;14:62. doi: 10.1186/s13321-022-00636-1 (PMC9476372; doi:10.1186/s13321-022-00636-1)
Supplement: Supplementary file 5 — Additional file 5. Cosine similarity values for pairs of GC-EI-MS spectra for each TMS derivative. [file 13321_2022_636_MOESM5_ESM.docx]

**Machine learning for identification of silylated derivatives from mass spectra**

Milka Ljoncheva ^†, ‡^, Tomaž Stepišnik ^∫, ‡^, Tina Kosjek ^†, ‡^, Sašo Džeroski ^∫, ‡, *^

*^†^ Jozef Stefan Institute, Department of Environmental Sciences, Jamova 39, 1000 Ljubljana, Slovenia*

*^∫^ Jozef Stefan Institute, Department of Knowledge Technologies, Jamova 39, 1000 Ljubljana, Slovenia*

*^‡^ Jozef Stefan International Postgraduate School, Jamova 39, 1000 Ljubljana, Slovenia*

**Additional file 5**

**Cosine similarity values for pairs of GC-EI-MS spectra for each TMS derivative**

| **CEC-TMS** | **RAW_min** | **BS_min** | **RAW vs BS_min** | **RAW vs BS_max** |
| --- | --- | --- | --- | --- |
| BPAF-TMS | 0.96570 | 0.96743 | 0.9666 | 0.99984 |
| DH-BP-TMS | 0.99634 | 0.99635 | 0.99532 | 0.99952 |
| 2APA-TMS | 0.40567* | 0.61693 | 0.45191* | 0.99966 |
| CLP-TMS | 0.95450 | 0.96080 | 0.96702 | 0.9994 |
| 3M5NC-TMS | 0.99144 | 0.99158 | 0.99125 | 1.00000 |
| 3MC-TMS | 0.99870 | 0.99870 | 0.99871 | 1.00000 |
| 44BP-TMS | 0.99151 | 0.99267 | 0.99151 | 0.99983 |
| HPP-TMS | 0.99526 | 0.99529 | 0.99494 | 0.99986 |
| 4HBP-TMS | 0.95110 | 0.95960 | 0.95170 | 0.99970 |
| 4NC-TMS | 0.99470 | 0.93140 | 0.93147 | 1.00000 |
| 4NG-TMS | 0.98150 | 0.98199 | 0.98137 | 0.99998 |
| 4NS-TMS | 0.86990 | 0.87151 | 0.86739 | 0.99992 |
| 4NP-TMS | 0.99920 | 0.99920 | 0.99891 | 0.99997 |
| 4OP-TMS | 0.99631 | 0.99665 | 0.99630 | 1.00000 |
| 5A3B17B-TMS | 0.98335 | 0.98335 | 0.98311 | 0.99992 |
| 5NG-TMS | 0.97614 | 0.97706 | 0.97610 | 1.00000 |
| 6BHP-TMS | 0.97350 | 0.98710 | 0.87493 | 0.97417 |
| 6MAM-TMS | 0.97241 | 0.92294 | 0.85121 | 0.99458 |
| 6NG-TMS | 0.55572 | 0.75368 | 0.55499 | 0.99923 |
| 8HQ-TMS | 0.99749 | 0.99886 | 0.99746 | 1.00000 |
| 9HF-TMS | 0.99950 | 0.99949 | 0.99916 | 1.00000 |
| 11AHD-TMS | 0.88172 | 0.94666 | 0.87879 | 0.97122 |
| 11HT-TMS | 0.82048 | 0.84894 | 0.66529 | 0.97490 |
| 1OHTHC-TMS | 0.92568 | 0.96954 | 0.96419 | 0.99960 |
| 11N9THC-TMS | 0.57328 | 0.94784 | 0.84188 | 0.99754 |
| E2-TMS | 0.93090 | 0.93103 | 0.92600 | 1.00000 |
| EE2-TMS | 0.91914 | 0.86014 | 0.47545* | 0.97808 |
| 17AHP-TMS | 0.81634 | 0.83103 | 0.54572 | 0.92389 |
| AA-TMS | 0.95802 | 0.96038 | 0.95796 | 1.00000 |
| AMP-TMS | 0.80340 | 0.80490 | 0.59033 | 0.99797 |
| PAA-TMS | 0.98931 | 0.99120 | 0.98992 | 0.99993 |
| BA-TMS | 0.99721 | 0.99869 | 0.99720 | 1.00000 |
| BZECG-TMS | 0.93830 | 0.94851 | 0.89206 | 0.99747 |
| BzPb-TMS | 0.97635 | 0.97635 | 0.97635 | 1.00000 |
| BP22BF-2TMS | 0.99420 | 0.99548 | 0.99463 | 0.99995 |
| BP24BF-2TMS | 0.98170 | 0.99001 | 0.97900 | 0.99985 |
| BPA-2TMS | 0.99700 | 0.99932 | 0.99788 | 0.99989 |
| BPAP-2TMS | 0.98630 | 0.98880 | 0.98636 | 0.99998 |
| BPB-2MTS | 0.98985 | 0.99430 | 0.98985 | 1.00000 |
| BPBP-2TMS | 0.94440 | 0.97044 | 0.94366 | 1.00000 |
| BPC-2TMS | 0.99550 | 0.99780 | 0.99603 | 0.99979 |
| BPCL-2TMS | 0.93270 | 0.95872 | 0.93637 | 0.99886 |
| BPE-2TMS | 0.99730 | 0.99825 | 0.99742 | 0.99998 |
| BPF-2TMS | 0.99290 | 0.99470 | 0.99340 | 0.99994 |
| BPFL-2TMS | 0.94610 | 0.97374 | 0.93430 | 0.99894 |
| BPM-2TMS | 0.96007 | 0.97164 | 0.96442 | 0.99986 |
| BPP-2TMS | 0.95154 | 0.98032 | 0.93817 | 1.00000 |
| BPPH-2TMS | 0.93264 | 0.95572 | 0.92458 | 0.99915 |
| BPS-2TMS | 0.88452 | 0.88936 | 0.88109 | 0.99990 |
| BPZ-2TMS | 0.94769 | 0.97523 | 0.94329 | 0.99876 |
| BD-TMS | 0.98605 | 0.94821 | 0.95083 | 0.99980 |
| BP26DM-2TMS | 0.97931 | 0.98543 | 0.97955 | 0.99992 |
| BHT-TMS | 0.70610 | 0.65651 | 0.66921 | 0.99994 |
| BuPb-TMS | 0.99150 | 0.99141 | 0.99117 | 0.99998 |
| CBC-TMS | 0.99691 | 0.99808 | 0.99241 | 0.99957 |
| CBD-TMS | 0.79430 | 0.80357 | 0.77775 | 0.99989 |
| CBDA-TMS | 0.72626 | 0.17776* | 0.09198 | 1.00000 |
| CBN-TMS | 0.99817 | 0.99877 | 0.99253 | 0.99895 |
| CBZ-TMS | 0.99563 | 0.99555 | 0.99116 | 0.99951 |
| CAT-TMS | 0.99730 | 0.99728 | 0.99728 | 1.00000 |
| CA-TMS | 0.99180 | 0.99275 | 0.99176 | 0.99993 |
| CLA-TMS | 0.99048 | 0.99052 | 0.99041 | 0.99998 |
| COD-TMS | 0.92495 | 0.86403 | 0.83099 | 0.99480 |
| THC-TMS | 0.74546 | 0.75612 | 0.72864 | 0.99951 |
| DF-TMS | 0.94290 | 0.94070 | 0.92732 | 0.99488 |
| BP8-TMS | 0.99468 | 0.99514 | 0.99261 | 0.99962 |
| ERY-TMS | 0.98660 | 0.98862 | 0.98590 | 0.99998 |
| E3-TMS | 0.61320 | 0.76623 | 0.47172* | 0.99703 |
| E1-TMS | 0.92030 | 0.93806 | 0.91174 | 0.99976 |
| EtPb-TMS | 0.98535 | 0.98552 | 0.98518 | 0.99999 |
| ETO-TMS | 0.70198 | 0.68205 | 0.68498 | 0.99983 |
| IB-TMS | 0.9943 | 0.99426 | 0.99424 | 1.00000 |
| IBuPb-TMS | 0.99846 | 0.99846 | 0.99843 | 0.99999 |
| IPrPb-TMS | 0.9908 | 0.9908 | 0.99068 | 1.00000 |
| KET-TMS | 0.94332 | 0.94533 | 0.93726 | 0.99838 |
| LAA-TMS | 0.88614 | 0.89453 | 0.88937 | 0.99980 |
| LLEU-TMS | 0.99301 | 0.93097 | 0.19120* | 0.23610* |
| LSER-TMS | 0.93808 | 0.99309 | 0.20496* | 0.27776* |
| LTYR-TMS | 0.23620* | 0.24113* | 0.23301* | 0.99943 |
| MCA-TMS | 0.86532 | 0.87740 | 0.86544 | 0.99987 |
| MAMP-TMS | 0.64650 | 0.72909 | 0.712990 | 1.00000 |
| MePb-TMS | 0.98570 | 0.98563 | 0.98557 | 1.00000 |
| MORPH-TMS | 0.80364 | 0.75554 | 0.65393 | 0.99798 |
| NAP-TMS | 0.99516 | 0.99573 | 0.99368 | 0.99974 |
| NX-TMS | 0.71481 | 0.42399* | 0.38158* | 0.99871 |
| OCA-TMS | 0.86140 | 0.76597 | 0.76215 | 0.99967 |
| PCA-TMS | 0.99122 | 0.99177 | 0.99133 | 0.99998 |
| PrPb-TMS | 0.98731 | 0.98718 | 0.98718 | 0.99999 |
| QA-TMS | 0.77030 | 0.71481 | 0.77088 | 1.00000 |
| RES-TMS | 0.99788 | 0.99789 | 0.99788 | 1.00000 |
| SA-2TMS | 0.99711 | 0.99712 | 0.99710 | 1.00000 |
| SA-TMS | 0.25956* | 0.19708* | 0.20060 | 1.00000 |
| SHA-TMS | 0.77778 | 0.77448 | 0.75270 | 0.99868 |
| STA-2TMS | 0.78030 | 0.85907 | 0.76395 | 0.98015 |
| STA-TMS | 0.98470 | 0.98466 | 0.98475 | 0.99996 |
| SFA-2TMS | 0.87792 | 0.81827 | 0.66323 | 0.98592 |
| SFA-TMS | 0.77374 | 0.82076 | 0.68291 | 0.99486 |
| SYR-TMS | 0.99899 | 0.99890 | 0.99886 | 1.00000 |
| THCA-TMS | 0.87845 | 0.85428 | 0.80438 | 0.99979 |
| T3HC-TMS | 0.87366 | 0.93768 | 0.87497 | 0.99948 |
| DHDPE-TMS | 0.99958 | 0.99960 | 0.92739 | 0.99895 |
| TCS-TMS | 0.99313 | 0.93220 | 0.99925 | 0.99990 |
| UA-TMS | 0.86660 | 0.72116 | 0.86631 | 0.99999 |

*-cosine similarity below the threshold.
