# Supplementary material for: Machine learning for identification of silylated derivatives from mass spectra
Source: J Cheminform. 2022 Sep 15;14:62. doi: 10.1186/s13321-022-00636-1 (PMC9476372; doi:10.1186/s13321-022-00636-1)
Supplement: Supplementary file 6 — Additional file 6. Structural classification of CEC-TMS derivatives. [file 13321_2022_636_MOESM6_ESM.docx]

**Machine learning for identification of silylated derivatives from mass spectra**

Milka Ljoncheva ^†, ‡^, Tomaž Stepišnik ^∫, ‡^, Tina Kosjek ^†, ‡^, Sašo Džeroski ^∫, ‡, *^

*^†^ Jozef Stefan Institute, Department of Environmental Sciences, Jamova 39, 1000 Ljubljana, Slovenia*

*^∫^ Jozef Stefan Institute, Department of Knowledge Technologies, Jamova 39, 1000 Ljubljana, Slovenia*

*^‡^ Jozef Stefan International Postgraduate School, Jamova 39, 1000 Ljubljana, Slovenia*

**Additional file 6**

#
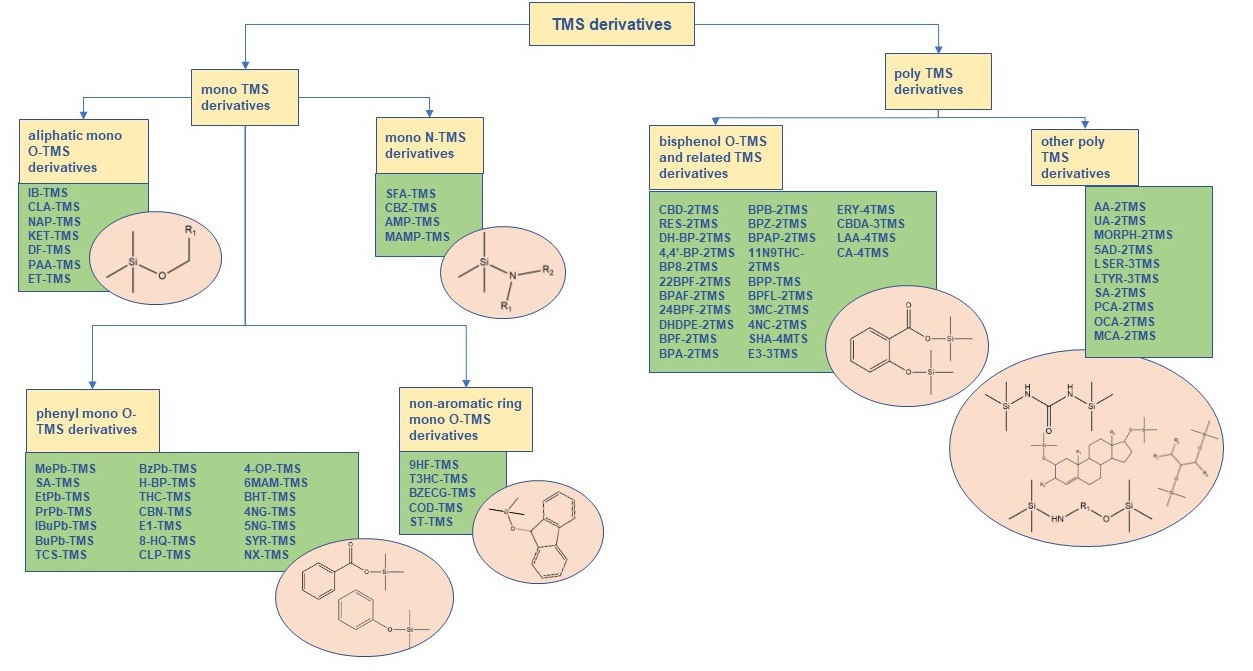
Structural classification of CEC-TMS derivatives
